# CRISPR/Cas9 system: recent applications in immuno-oncology and cancer immunotherapy

**DOI:** 10.1186/s40164-023-00457-4

**Published:** 2023-11-14

**Authors:** Chen Chen, Zehua Wang, Yanru Qin

**Affiliations:** Department of Oncology, The First Affiliated Hospital of Zhengzhou University, Zhengzhou, China

**Keywords:** CRISPR/Cas9, Adoptive cell therapy, Cancer immunotherapy, Gene editing, Target discovery

## Abstract

Clustered regulatory interspaced short palindromic repeats (CRISPR)/CRISPR-associated protein 9 (Cas9) is essentially an adaptive immunity weapon in prokaryotes against foreign DNA. This system inspires the development of genome-editing technology in eukaryotes. In biomedicine research, CRISPR has offered a powerful platform to establish tumor-bearing models and screen potential targets in the immuno-oncology field, broadening our insights into cancer genomics. In translational medicine, the versatile CRISPR/Cas9 system exhibits immense potential to break the current limitations of cancer immunotherapy, thereby expanding the feasibility of adoptive cell therapy (ACT) in treating solid tumors. Herein, we first explain the principles of CRISPR/Cas9 genome editing technology and introduce CRISPR as a tool in tumor modeling. We next focus on the CRISPR screening for target discovery that reveals tumorigenesis, immune evasion, and drug resistance mechanisms. Moreover, we discuss the recent breakthroughs of genetically modified ACT using CRISPR/Cas9. Finally, we present potential challenges and perspectives in basic research and clinical translation of CRISPR/Cas9. This review provides a comprehensive overview of CRISPR/Cas9 applications that advance our insights into tumor-immune interaction and lay the foundation to optimize cancer immunotherapy.

## Backgrounds

Clustered regulatory interspaced short palindromic repeats (CRISPR)/CRISPR-associated protein 9 (Cas9) is essentially an adaptive immunity weapon in prokaryotes to against foreign DNA, which is adapted as a groundbreaking genome-editing tool in eukaryotic cells [1]. CRISPR/Cas9 shows superior flexibility, scalability, and operability than other gene editing technologies, such as Zine finger nucleases (ZFNs) and transcription activator-like effector nucleases (TALENs) [2–4] **(**Table-1**)**. CRISPR/Cas9 system consists of a CRIPSR single guide RNA (sgRNA) and a Cas9 nuclease. When the sgRNA recognizes the target DNA sequence, it instructs the Cas9 protein to cut the DNA and create a double-strand break (DSB), followed by the silence of a selected gene [5]. The damaged DNA is then repaired by either non-homologous end joining (NHEJ) or homology directed repair (HDR), achieving the purpose of gene knockout or target gene replacement [5, 6]. Simply changing the sgRNA sequence allows the CRISPR/Cas9 to target any part of the desired genome, prompting the development of pooled, genome-scale CRISPR libraries [7, 8].

**Table 1:** Comparison of genome editing technologies

	ZFN	TALEN	CRISPR
**Length of target sequence**	9-12 bp	14-20 bp	20 bp + PAM
**Target DNA recognition**	DNA-protein interaction	DNA-protein interaction	DNA-RNA interaction
**Target site**	Single	Single	Multiple
**Nuclease**	Fok1	Fok1	Cas9
**Efficacy**	Moderate	Moderate	High
**Specificity**	Low	Moderate	High
**Toxicity**	High	Moderate	Moderate
**Design difficulty**	Complicated	Complicated	Simple
**Clinical trial**	Moderate	Low	High
**Disadvantage**	Toxic; limited site selection; expensive	Difficult to deliver due to the large size	Target DNA must have an upstream PAM; off-target effects

The majority of CRISPR screening utilizes in vitro models that cannot fully reflect the complex cellular and microenvironment signals. However, high-throughput CRISPR/Cas9-mediated in vivo screening, facilitated by Cas9-expressing mice, is a powerful platform to determine driver genes that orchestrate immune cell function in the tumor microenvironment (TME) as well as to unravel novel cancer-mediated, immune-inhibitory targets [9–12]. CRISPR screens allow for mapping tumor-immune interaction to identify regulators that synergize or alleviate immune escape, underling the fundamental of next-generation cancer immunotherapy.

Cancer immunotherapy has ushered in a new field for the treatment of various malignancies, aiming to redirect or stimulate the host’s immune system to attack proliferating tumor cells. Importantly, recent development of adoptive cell therapy (ACT) shows tremendous potential to combat tumors by using genetically modified T cells [13]. ACT generally includes chimeric antigen receptor (CAR) T cell therapy, T cell receptor (TCR) T cell therapy, and tumor-infiltrating lymphocytes (TIL) therapy. However, the success of ACT is usually hampered by off-target toxicity and limited efficacy [14]. This phenomenon is more significant in solid tumors than blood malignancies. Recently, the introduction of CRISPR/Cas9, a versatile platform for genetic engineering, might circumvent the above obstacles, paving the way for the next-generation adoptive cellular therapy to precisely target the intended cells and alleviate adverse events.

In this review, we introduce the mechanism of CRISPR/Cas9 genome editing, review applications of CRISPR in generating mouse models and organoids, emphasize the progress of CRISPR/Cas9 screening for target discovery, demonstrate the potential of CRISPR/Cas9 to advance T-cell-based therapy, present the next-generation CRISPR editing approaches, and finally discuss challenges and prospects in cancer research and therapy of CRISPR/Cas9. Given its outstanding role in biomedical science, CRISPR/Cas9 will deepen our understanding of the immune response under tumor pressure, identifying novel immune-oncology targets and boosting the therapeutic potential of ACT.

### CRISPR/Cas9 system: structure and mechanism

**Fig. 1:**
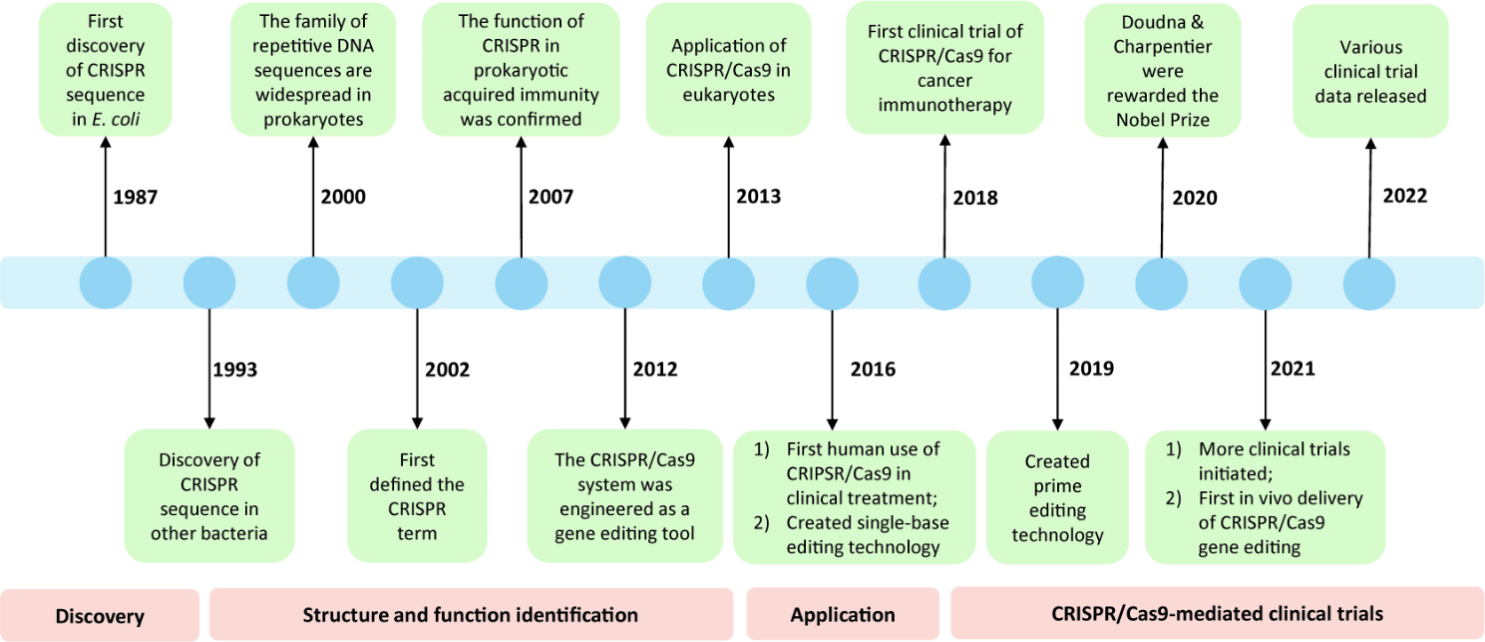
The timeline of CRISPR/Cas9

In the late 1980s, CRISPR was first discovered in bacterial adaptative immunity against foreign genetic elements through an RNA-guided DNA cleavage system over three steps: acquisition, transcription, and interference [15, 16]. Since then, researchers have investigated the structure and function of CRISPR/Cas9 to establish site-specific gene editing strategies in humans for clinical applications (Fig. 1). The first described system is the type-II CRISPR/Cas9 genome editing tool from *Streptococcus pyogenes* Cas9 (SpCas9). When encountering a foreign DNA invasion, a DNA segment from the invader was integrated into the host CRISPR locus as a spacer [17]. This locus is then transcribed to CRISPR-derived RNA (crRNA) that attaches to a transactivating crRNA (tracrRNA). The crRNA: tracrRNA duplex constitutes a single gRNA (sgRNA), which loads onto the Cas9 protein to assemble a ribonucleoprotein (RNP) complex. The sgRNA guides Cas9 to create a site-specific DSB at the target DNA site complementary to the crRNA sequence [18]. Overall, the CRISPR/Cas9 system comprises two critical components: a Cas9 nuclease and a sgRNA.

**Fig. 2:**
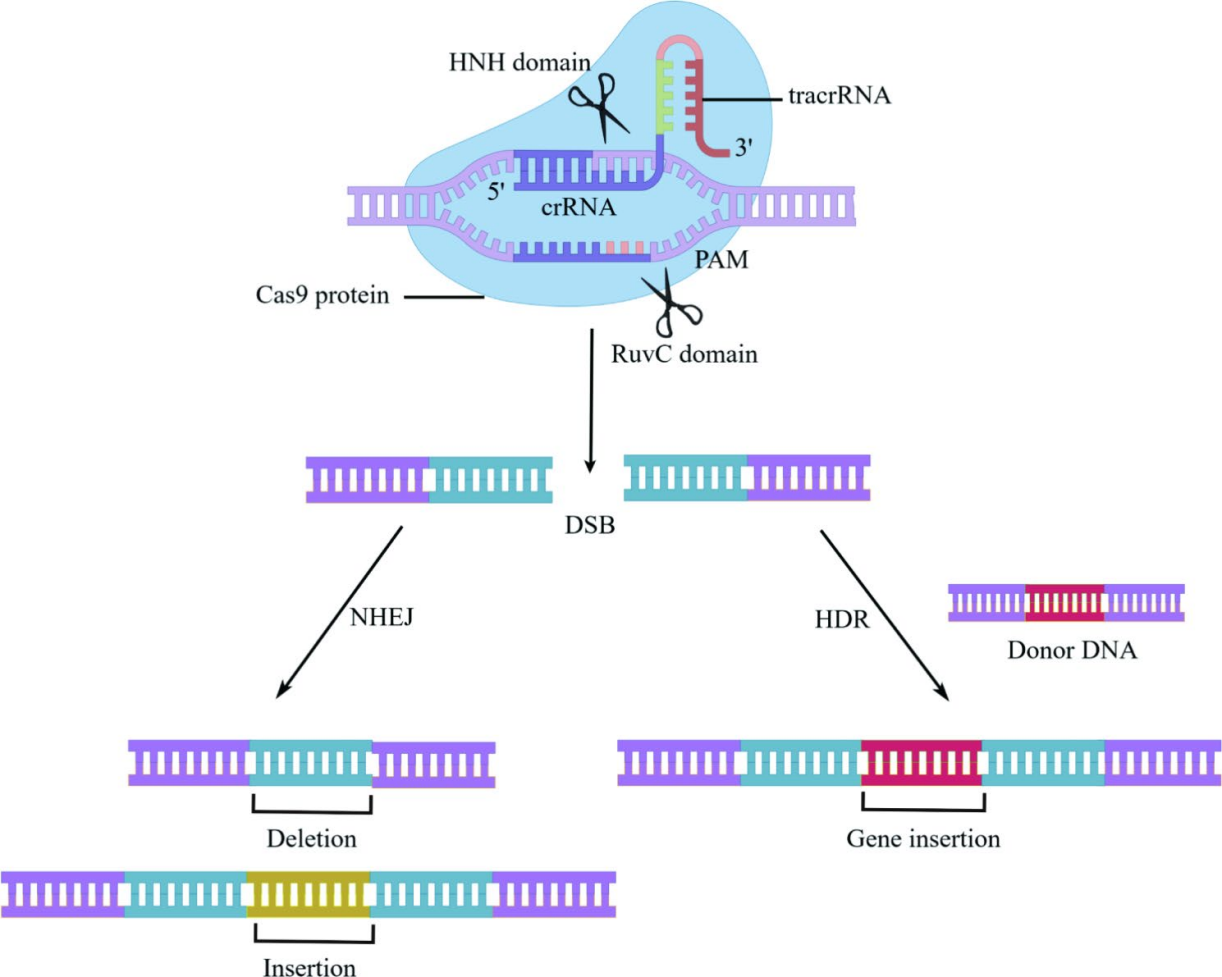
Mechanism of CRISPR/Cas9-mediated DSB and subsequent repair. When the sgRNA forms base pairs with target DNA, two endonuclease domains of Cas9 (the HNH and RuvC domain) create a site-specific double-strand break (DSB) into DNA. Subsequently, DSBs are repaired by non-humongous end joining (NHEJ) or homology directed repair (HDR) mechanism

Altering the target DNA only needs designing different sgRNAs. The first 20 bp of sgRNA at the 5’ end recognizes a specific sequence via Watson-Crick base pairing, while the 3’ end binds to the Cas9 nuclease that introduces a DSB into the target DNA at the upstream of the protospacer adjacent motif (PAM). There are two prerequisites for sgRNA-Cas9 coupling to bind and cleave target DNA: (1) site-specific complementarity between the 20-nucleotide of sgRNA and the target DNA, (2) the existence of PAM (5’-NGG for SpCas9) adjacent to the target sequence [19]. Without PAM, even if entirely complementary to sgRNA, Cas9 still cannot cleave the target DNA [20]. Of note, when the target DNA containing the PAM component complements sgRNA to form the R-loop (RNA-DNA hybrid), the target sequence would be cleaved by two distinct domains of Cas9 nuclease: the HNH and RuvC-like domains [21–23]. Subsequently, DSBs are repaired by either NHEJ or HDR. NHEJ is an error-prone repair mechanism that usually creates random insertions or deletions (indels), leading to frameshift mutations or genome rearrangements [24]. HDR utilizes a homologous DNA template to recognize DSB and insert the donor DNA at CRISPR cleavage sites [25, 26]. More recently, the CRISPR/Cas9 plays a pivotal role in genome modification. Fusion of Cas9 protein mutant dCas9 (endonuclease-deficient Cas9) to activator or repressor could regulate gene transcription, respectively mediating CRISPR activation (CRISPRa) and CRISPR interference (CRISPRi) [27]. Different RNA-guided Cas9 systems have been developed and tested in various clinical trials. In this review, we mainly focus on the impacts of CRISPR/Cas9 gene editing on immuno-oncology, providing novel insights for cancer immunotherapy. Schematic illustration of molecular mechanism of CRISPR/Cas9 has been shown in Fig. 2.

### Immuno-oncology: CRISPR/Cas9 in Tumor modeling

**Fig. 3:**
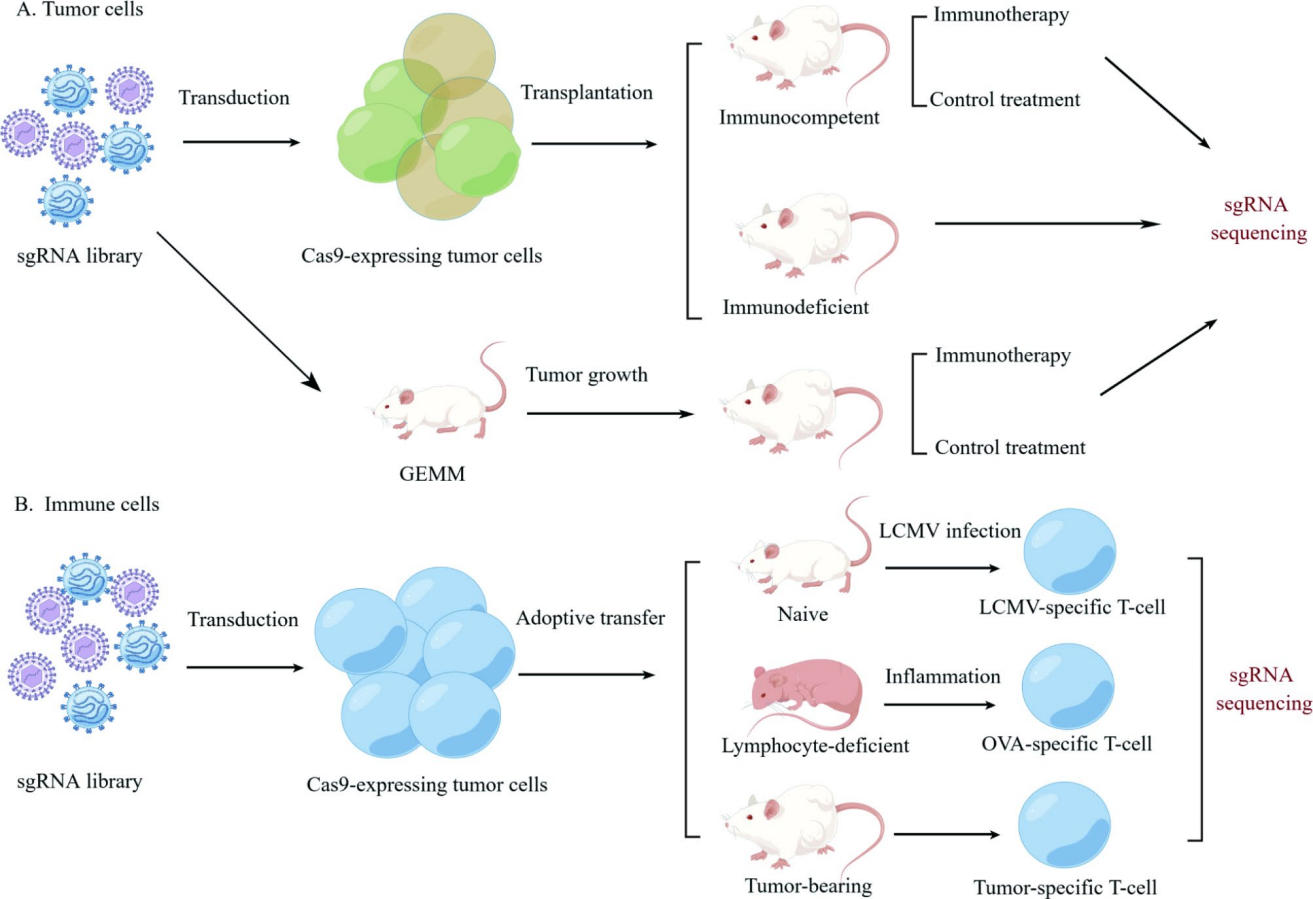
In vivo CRISPR modeling in tumor and immune cells. **A**. Tumor cells. Cas9-expressing tumor cells transduced with sgRNA library are implanted into immunocompromised or immunocompetent mice that model the absence or presence of immune surveillance. Immunocompetent mice were treated with different immunotherapies to discover genes reflecting drug-responsiveness. **B**. Immune cells. A pooled CRISPR sgRNA library was transduced into T cells that express Cas9 and transgenic T cell receptor (TCR) before being adoptively transferred to mouse models. Antigen-specific T cells isolated from mice are sequenced to compare their sgRNA abundance with that of pre-transferred T cells, revealing T cell response in the context of infection, inflammation, and tumor

Intracellular and TME signals that coexist in vivo play a critical role in immune cell activation, proliferation, and function, which cannot be easily imitated in vitro models. Thereby, CRISPR technology facilitates a process from in vitro to in vivo. In vivo CRISPR modeling (Fig. 3), with the aid of Cas9-expressing or dCas9-expressing mouse models [28–30], has unique advantages in assessing tumor proliferation and response to T-cell based therapy [31]. Alternatively, organoids are advanced in vitro 3D models and can complement in vivo models to better simulate the TME.

### CRISPR in vivo modeling of Tumor cells

Various CRISPR-engineered models have been developed to identify factors manipulating immune pressure in tumor models. Heterotopic transplantation of tumor cells is a promising strategy to evaluate tumorigenic potential and response to immunotherapy. Cas9-expressing tumor cells transduced with sgRNA library are implanted into immunocompromised or immunocompetent mice that simulate the absence or presence of immune surveillance [32–34]. Immunocompetent mice are treated with immune checkpoint blockade (ICB), adoptive T-cell transfer, or cancer vaccines. Subsequently, tumor cells from immunodeficient, immunocompetent, and immunotherapy-treated mouse models are extracted to perform sgRNA sequencing. These in vivo CRISPR models unravel genes involved in complicated interactions between tumor cells, T cells, and the TME, greatly expanding upon studies focused on immuno-oncology interplay in vitro [35, 36].

In addition, CRISPR-mediated genetically engineered mouse models (CRISPR-GEMMs) have been established, wherein orthotopically implanted tumors form in an original immune microenvironment that closely models human tumorigenesis [37]. In this case, the CRISPR library contains a tissue-specific promoter, sgRNA expression cassettes, and another sgRNA targeting *TP53*. The pooled library is then transduced into inducible Cas9-expressing GEMMs; thereby, sgRNA-mediated gene knockout occurs within a tissue-specific or cell type-specific manner. The GEMMs are monitored for tumor growth by bioluminescence and treated with ICI or a control followed by sgRNA sequencing. The success of CRISPR-GEMMs relies on how to deliver perturbation reagents to target cells efficiently, which greatly vary in different organs; hence, the current application of CRISPR-GEMMs is limited to brain, lung, and liver cancers [28, 37, 38]. Overall, constructing organ-specific tumor models is expected to identify tumor type-specific immune response, paving the way for precision immuno-oncology [39].

### CRISPR in vivo modeling of immune cells

In vivo CRISPR modeling, a pooled CRISPR sgRNA library was transduced into T cells that express Cas9 and transgenic T cell receptor (TCR) before being adoptively transferred to mouse models. Antigen-specific T cells isolated from mice are sequenced to compare their sgRNA abundance with that of pre-transferred T cells, revealing T cell response in the context of infection, inflammation, and tumor.

Mouse infection models have been used to unravel the functional differentiation of effector T cells [40]. For example, naïve mice infected with lymphocytic choriomeningitis (LCMV) are injected with Cas9-expressing CD4^+^ T cells in vivo metabolic CRISPR screening, which identified ETNK1 and PCYT2 as regulators in the T follicular helper (T_FH_) cells versus T_H_1 cell fate decision [41]. Mouse inflammation models are used to identify genes that regulate adaptive immune response in vivo [42, 43]. The sgRNA library-transduced CD4^+^ T cells are implanted into lymphocyte-deficient *Rag1*^*−/−*^ mice that are immunized with ovalbumin (OVA) to induce lung inflammation. In this case, MTHFD2 is identified as a metabolic checkpoint controlling the proliferation and inflammatory function of lung-derived T cells [43]. CRISPR/Cas9 technology is also applied for tumor-bearing mice, identifying regulators to improve CD8^+^ T cell effector function. In vivo CRISPR-screening for mouse CD8^+^ T cell fitness has been conducted in orthotopically implanted GL261 glioblastoma and E0771-OVA triple-negative breast cancer models. In these screenings, DHX37, PDIA3, and MGAT5 were identified as negative regulators of T cell response [44, 45].

### CRISPR-engineered organoids in Tumor modeling

In addition to animal models, organoids are self-assembling, three-dimensional (3D) cellular structures that retain critical features of the original tissue in vitro. Patient-derived organoids (PDOs) emerged as a novel preclinical model closely resembling patients’ tumors, which fills the conventional gaps in patient-derived cancer cells (PDCs) and patient-derived xenografts (PDXs). CRISPR allows the engineering of PDOs by introducing cancer gene mutations into normal organoids.

Organoids could be derived from embryonic stem cells, induced pluripotent stem cells (iPSCs), and somatic stem cells (SSCs). Stem cells from different tissues are isolated and cultivated in a 3D culture. Given the ability of self-renewal while retaining the genotype and phenotype of their parent tissues, stem cells constantly proliferate, differentiate, and form the organoid in ex vivo. With recent technological advances in 3D culture systems, CRISPR fosters the generation of genetically predefined tumor models to identify gene functions and tailor precision medicine in cancer treatment [46]. Lo et al. constructed gastric cancer organoid models with or without ARID1 mutations engineered by CRISPR/Cas9, revealing context-dependent roles of ARID1 in the early transformation of gastric cancer [47]. Two groups of Sato and Clevers transformed healthy human colon organoids into their cancerous counterparts by recapitulating the classic ‘Vogelgram’ sequence of colon cancer by CRISPR/Cas9 gene editing [48, 49]. Drost et al. applied CRISPR/Cas9 technology to knockout DNA repair genes to model mismatch repair (MMR)-deficient colon cancer organoids, revealing the mutational signatures underlying cancer initiation and progression [50]. Similar two studies confirmed the feasibility of using CRISPR/Cas9 technology to knockout tumor suppressor genes in small cell lung cancer (SCLC) and breast cancer organoid models, which helps explore the pathogenesis and drug responses [51, 52].

Collectively, CRISPR-engineered organoids not only overcome the limitations of 2D cell culture but also complement animal models for gene interrogation. More importantly, tumor organoids can be used to verify findings obtained from other model systems. Of note, more studies of CRISPR-edited organoids are still required in the immune-oncology intersection.

### Immuno-oncology: CRISPR/Cas9 in target discovery

CRISPR screening is a powerful platform for biological discovery, using genome-scale sgRNA libraries for high-throughput identification of drug targets and functional genes [53, 54]. CRISPR screens take full advantage of the efficiency and flexibility of CRISPR/Cas9 genome editing by enabling gene knock-out at the DNA level [11, 18]. Compared with RNA interference (RNAi)-mediated loss-of-function screening, CRISPR/Cas9 shows higher sensitivity, less non-targeted interference, and slighter off-target effects [55].

There are two formats of CRISPR screening: pooled and arrayed [56]. In a pooled CRISPR screen, a sgRNA library is introduced into a bulk population of cells, such that individual cells undergo distinctive sgRNA-mediated genetic perturbation. These sgRNAs are delivered by lentiviral or retroviral vectors and integrated into the DNA of the targeted cell. The gene-edited cells proliferate under selective pressure, such as drug treatment, cell competition, or viral infection. As engineered genetic perturbations affect the fitness of cells, competition exists among these cells for survival. The sgRNAs retained in the pool after the challenge are counted and specified with high-throughput sequencing or imaging technology. In arrayed CRISPR screens, cells with different sgRNA-mediated perturbations are introduced in individual wells of a multi-well plate and remain physically separated. As gene perturbations in individual reaction compartments are predefined, read-outs do not need any sequencing, such as proteomic, metabolomics, or imaging profiling in arrayed screens. Even though arrayed screens are primarily used for validation and mechanistic investigation, they are more labor-intensive and have lower throughput than pooled arrays; thereby, studies extensively apply pooled screens for discovery. CRISPR has unraveled several molecules in the immune-oncology field. Therefore, we list the recent findings in CRISPR/Cas9-mediated immune-oncology target discovery (Table 2).

**Table 2:** CRISPR/Cas9-mediated target discovery in the immuno-oncology

Location	Target	Biological function	Genome-editing technology	Effects after CRISPR-engineering	Reference
**Tumor cell**	PTPN2	A phosphate mediating IFN-γ sensing	CRISPR-KO	PTPN2 KO increases antigen presentation and anti-tumor toxicity.	[36]
	ASAF1	A regulator of drug-sensitivity	CRISPR-KO	ASAF1 KO prompts M1-type macrophage polarization and potentiates T-cell activation	[35]
	Cop1	A modulator recruiting M2-type macrophage	CRISPR-KO	Cop1 KO decreases immune escape and enhances ICI efficacy	[36]
	KEAP1	Drug-resistance gene	CRISPR-KO	KEAP1 KO allows tumor cells to proliferate without MAPK signaling.	[57]
**Immune cell**	REGNASE1	Metabolism-related gene	CRISPR-KO	REGNASE1 KO enhances the accumulation of tumor-specific T-cell	[58]
	CARM1	Epigenetic enzyme	CRISPR-KO	CARM1 KO enhances anti-tumor immunity and sensitizes resistant tumors to ICI	[59]
	FLI1	Transcription factor	CRISPR-KO	CD8 + T-cells deleting Fli1 exert a more protective immunity.	[60]
	CBAF and INO80 complex	A regulator of T-cell exhaustion	CRISPR-KO	CBAF and INO80 complex KO prolongs T-cell persistence.	[61]
	FAM49B	Negative regulators of T-cell response	CRISPR-KO	FAM49B KO prompts T-cell activation.	[62]
**Tumor-immune interaction**	PRC2	A negative regulator of IFN-γ-induced MHC-1 expression	CRISPR-KO	PRC2 KO upregulates MHC-1 expression and enhance tumor recognition by immune cells.	[63]
	CMTM6	A positive regulator of IFN-γ-induced PD-L1 expression	CRISPR-KO	CMTM6 KO downregulates PD-L1 expression.	[64]
	TRAF3	A negative regulator for MHC-1 expression	CRISPR-KO	TRAF KO upregulates MHC-1 expression.	[65]
	SIGLEC	Glycan-binding immune receptor	CRISPR-KO	Blocking CD34-SIGLEC7 interplay makes tumor cells more vulnerable to immune cell attack.	[66]

Abbreviations. KO, knock out

### Target discovery in Tumor cells

Tumor evolves various mechanisms to occur immune escape, comprising impaired antigen presentation, activation of oncogenic signals, reduced responsiveness to IFN-$\gamma$, and upregulated drug-resistance genes [57]. Large-scale CRISPR Cas9 genomic screening is a powerful tool for detecting cancer-specific immune-inhibitory targets that drive tumor evolution.

Perturbating candidate molecules in the signaling pathway to ascertain their immunological function paves the way to enhance cancer immunotherapy. For example, phosphatase PTPN2 was identified as a novel immunotherapeutic target in the first in vivo CRISPR screening in tumor cells. PTPN2 deletion was confirmed to sensitize melanoma cells to ICB [32]. PTPN2 knockout has been shown to increase antigen presentation and T cell anti-tumor toxicity [36]. Similarly, in vivo epigenetic CRISPR screen discovered ASF1A as a critical regulator of lung adenocarcinoma sensitivity to immunotherapy [35]. Loss of ASF1A in lung cancer cells facilitates M_1_-type macrophage polarization by upregulating GM-CS expression and potentiates T cell activation via synergizing with anti-PD1 treatment. This provides a rationale for the combinatorial strategy of ASF1A inhibition and ICB therapy. In vivo CRISPR screens identified Cop1 as a modulator to recruit immunosuppressive macrophages that drive immune evasion, which is a potential target in triple-negative breast cancer to improve the efficacy of ICB [36].

Another application is identifying drug-resistant genes by integrating CRISPR screening and drug perturbation [58]. Despite the wide use of tyrosine kinase inhibitors (TKIs) in cancer treatment, most patients still fail to respond to them. CRISPR Cas9 knockout screening in lung cancer indicated that KEAP1 deletion alters cell metabolism, allowing cancer cell survival in the presence of RTK/Ras/MAPK pathway inhibitors [59]. Overall, loss-of-function screens help to discover drug-resistant genes to assist in treatment selection.

### Target discovery in immune cells

T cell function, expansion, and persistence are essential for anti-tumor immunity [60]. Regulators of T cell quality and quantity are potent targets to enhance immunotherapy. CRISPR/Cas9-mediated screening in T cells enables high-throughput identification of target genes that modulate T-cell behaviors, which can be achieved via loss-of-function (CRISPR knockout, CRISPR interference) or gain-of-function (CRISPR activation) [61].

The success of cancer immunotherapy is highly dependent on effector activity of antigen-specific T cells [62]. Wei et al. [63]. revealed metabolism-associated genes that can be reprogrammed to enhance T cell-mediated anti-tumor activities. In the B16-OVA melanoma models, the deficiency of ribonuclease REGNASE1 was proven to increase the infiltration of tumor-specific T cells within the tumor. CARM1, an epigenetic enzyme identified by CRISPR screening, exerts a dual role on both tumor cells and cytotoxic T cells to impede anti-tumor immune response [64]. Therefore, CARM1 inactivation elicits potent immunity while sensitizing resistant tumors to immunotherapy. CRISPR screen offers a platform to identify reciprocal regulators that simultaneously inhibit tumor growth and enhance T-cell functionality. In vivo T-cell CRISPR screening platform identified Fli1 as a transcription factor restraining effector T cell differentiation [65]. CD8^+^ T cells deleting Fli1 exert a more protective immunity against tumors. Likewise, T-cell exhaustion limits the efficacy of immunotherapy. BAF and INO80 chromatin remodeling complex was shown to restrict T-cell persistence with the genome-wide CRISPR screening [66]. Moreover, a genome-wide CRISPR screening discovered a critical regulator, FAM498, hampers T cell activation by inhibiting Rac activity and modulating cytoskeletal remodeling [67].

Regulatory T (T_reg_) cells are a specialized subset of CD4^+^ T cells that act to maintain self-tolerance and immune homeostasis. By incorporating CRISPR screens and single-cell sequencing, HIVEP2 and SATB1 coregulate another gene network essential for T_reg_-mediated immunosuppression, which can be targeted to design T_reg_-based immunotherapy [68]. These findings together show the robustness of CRISPR/Cas9 screening for identifying novel targets for immunotherapy. Applications of CRISPR screening in other immune cells, such as NK cells, B cells, dendritic cell, and macrophages will further deepen our understanding of immune regulatory networks.

### Surface protein of tumor-immune interaction

Deletion of MHC-I-mediated antigen presentation could impede CD8^+^ T cell activation, prompting tumor cells to evade T cell-mediated killing [57]. PRC2, a negative regulator of IFN-$\gamma$-mediated MHC-I expression on tumor cells, was identified by a fluorescence-activated cell sorting (FACS)-based CRISPR screen [69]. IFN-$\gamma$ signaling also affects the expression of PD-L1 that subsequently binds to PD-1 expressed by tumor-specific T cells. A CRISPR screen uncovered that CMTM6 maintains IFN-$\gamma$-mediated PD-L1 expression on tumor cells to prompt immune evasion [70]. TRAF3 was screened as a negative regulator for MHC-I expression but not for PD-L1 expression; thereby, targeting TRAF3 upregulates MHC-I expression to potentiate ICB efficacy [71]. SIGLECs, a family of glycan-binding receptors on the surface of immune cells, mediate tumor-immune interactions and are potential targets to enhance immune surveillance in vitro [72, 73]. A genome-wide CRISPR screen revealed a specific cell-surface glycan ligand (CD34) on leukemia cells that binds to SIGLEC7 protein. Blocking this interplay makes tumor cells more vulnerable to immune cell-induced lysis [57]. The application of CRISPR screens discovering ligand-receptor pairs underlying tumor-immune interactions provides novel insights into modulating the TME.

### CRISPR/Cas9 advances adoptive cell therapy

**Fig. 4:**
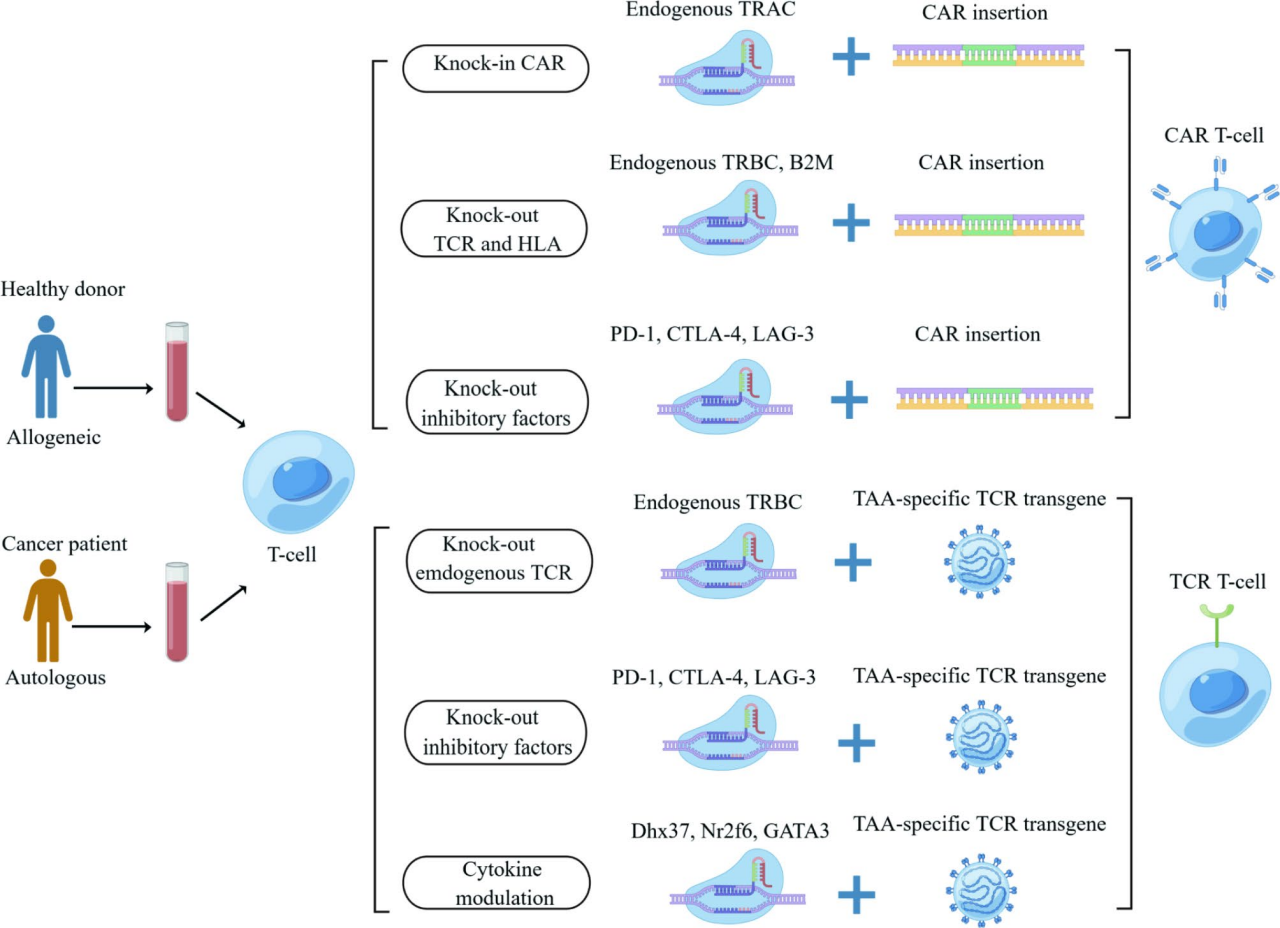
CRISPR/Cas9 engineering for adoptive T cell therapy. CRISPR-engineered T cells from either allogeneic or autologous T cells hold the promise to enhance the efficacy and reduce the toxicity of TCR T-cell and CAR T-cell therapy via several mechanisms, including endogenous TCR disruption, TCR and HLA deletion, immunosuppressive factor knock-out, and cytokine modulation. Abbreviations: TAA, tumor-specific antigen; TCR, T cell receptor; CAR, chimeric antigen receptor

ACT shows tremendous potential in treating hematologic malignancies [13]. However, several challenges preclude ACT from reaching its full potential, involving T-cell exhaustion, immunosuppressive TME, off-target toxicity, and poor quality of T-cell manufacture [74, 75]. The emergence of CRISPR/Cas9 sparked a hope to reinvigorate CAR T-cell and TCR T-cell therapy in the past few years [76] (Fig. 4).

### Engineering “armored” T cells

**Fig. 5:**
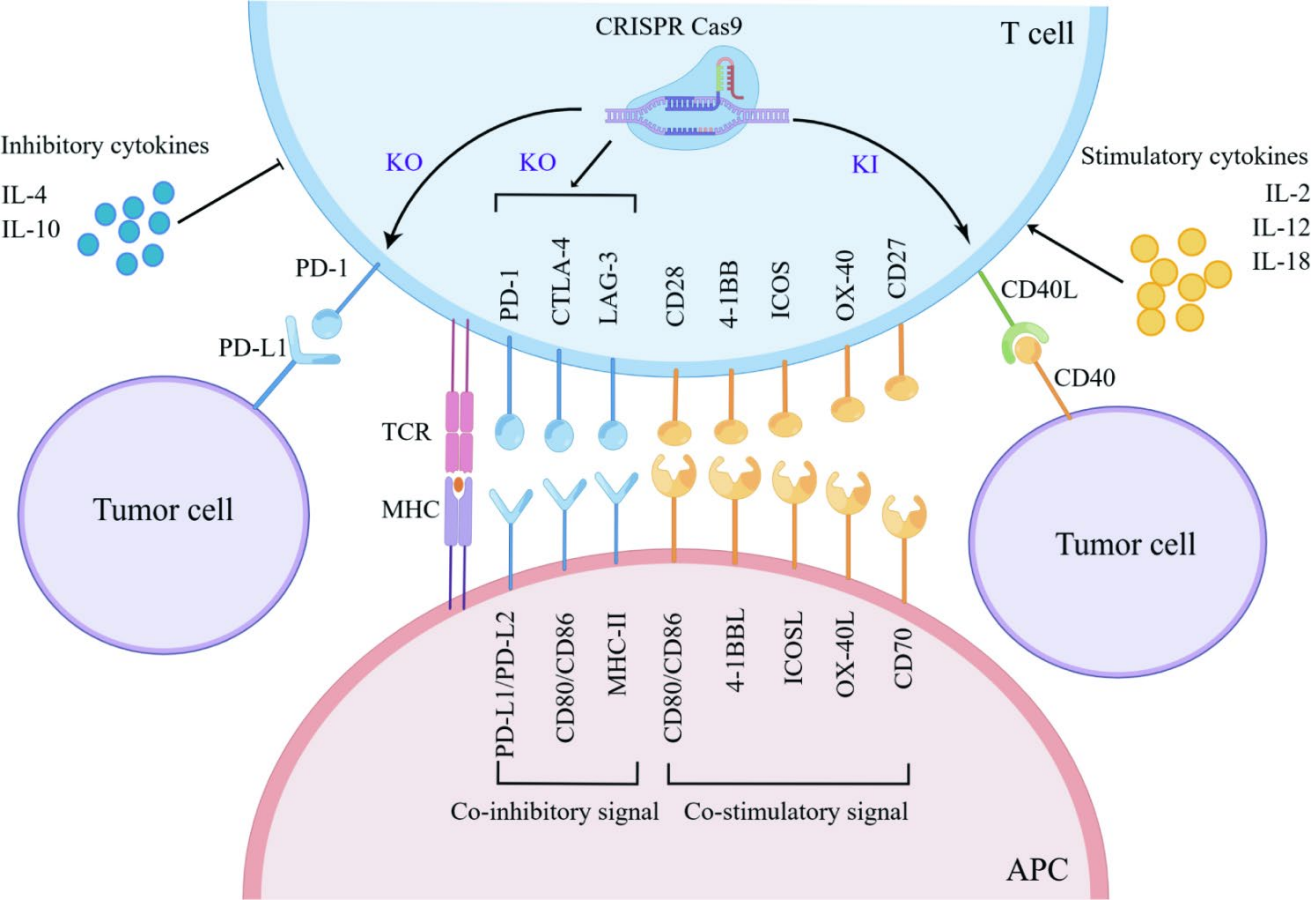
Stimulatory and inhibitory factors of T-cell activation. Modulating co-stimulatory signals and related cytokines is essential for T-cell activation. Conversely, eliminating inhibitory signals using CRISPR/Cas9-mediated gene knockout, such as PD-1 and CTLA-4, could make T-cell-based therapy more potent. Abbreviations: APC, antigen-presenting cell; KO, knock-out; KI, knock-in; TCR, T cell receptor

For T-cell activation, both a primary signal (TCR-MHC) and a secondary co-stimulation signal are required. TCR signaling triggers cytokine secretion essential for modulating CD8^+^ T-cell function [77]. Also, the balance between co-stimulation and co-suppression signals affects T-cell persistence for effective immune surveillance. Therefore, engineered T cells can “arm” themselves by CRISPR/Cas9 knock-in to secrete immune stimulatory cytokines or express ligands that improve the efficacy of ACT **(**Fig. 5**)**.

Besides CD28 and 4-1BB, many co-stimulatory domains have been incorporated into CAR T-cells, including CD27, OX40, and ICOS, giving rise to superior anti-tumor effect and prolonged T-cell persistence [78–81]. Studies have engineered CAR T-cells to constitutively express CD40L that binds to CD40-expressing tumor cells, inducing a direct cytotoxicity effect and circumventing immune escape [82, 83]. After T-cell adoptive transfer, CD40/CD40L interaction activates antigen-presenting cells (APC) via the NF-$\kappa$B pathway, enhances the recruitment of immune effectors, and mobilizes endogenous tumor-recognizing T cells. Furthermore, CRISPR/Cas9-mediated CD40L-armed oncolytic therapy allows the TME to occur in similar immunological processes as above, driving a persistent immune response in the pancreatic ductal adenocarcinoma (PDAC) mice model [84]. These provide a rationale for armoring T cells with CD40L by CRIPSR to optimize cancer immunotherapy.

Alternatively, cytokines are irreplaceable as the third signal outside TCR engagement and co-stimulation. CAR T-cells have been engineered to express IL-12 constitutively with CRISPR/Cas9, enhancing T-cell functionality and attracting macrophages to disrupt TNF-$\alpha$-mediated antigen-loss of tumor cells. Once encountering IL-12-secreting CAR T-cells, M_2_-type macrophages and myeloid-derived suppressor cells (MDSCs) lose their inhibitory ability in the TME [85]. Membrane-bound IL-15 CAR T-cells trigger STAT5 signaling critical for prompting anti-tumor activity and reversing T-cell energy [86]. Also, IL-18 is critical to increase IFN-$\gamma$ production and prompt CAR T-cell proliferation [87]. These stimulatory cytokines are usually transduced with viral vectors, while overexpression of cytokines may hamper T-cell cytotoxicity. Therefore, specific knock-in via CRISPR/Cas9 enables cytokines to express under the control of endogenous promoters. Another weapon is knocking out transcriptional factors (TFs) that inhibit cytokine secretion, such as GATA3 [88]. By contrast, CRISPR-mediated DHX37 ablation is reportedly to downregulate IL-6 secretion and enhance T-cell function [44]. AP-1 is another TF hamstrung by intracellular NR2F6. CRISPR knockout of NR2F6 has been shown to improve IFN-$\gamma$ secretion and exert a synergistic effect in combination with PD-1 blockade [89]. Overall, the enhanced T-cell proliferation and persistence using CRISPR/Cas9 gene editing has emphasized the importance of co-stimulatory domains and cytokines.

### Eliminating “immune brakes”

Resistance to ACT is motivated through T-cell-intrinsic and T-cell-extrinsic mechanisms. Extrinsic resistance comprises loss of target antigen and acquisition of genetic alterations. Conversely, intrinsic resistance develops when the transduced T-cells fail to kill tumor cells where the TME harbors an abundance of immunosuppressive cells, molecules, signaling cascades, and metabolic restrictions [90]. Hence, adoptive transferred T cells can be engineered to eliminate “immune brake” effects using CRISPR **(**Fig. 5**)**.

The application of ACT in solid tumors is usually impeded by T-cell dysfunction led by the upregulation of co-inhibitory signals, thereby removing these inhibitory molecules (PD-1, CTLA-4, LAG3) represents a first tractable strategy [91–94]. The most widely explored example is PD-1 knockout via CRISPR to reinvigorate CD8^+^ T cell effector function, which has been successfully validated in multiple pre-clinical models [92, 95–99] and clinical trials [100, 101]. CRISPR/Cas9-mediated double knockout of PD-1 and CTLA-4 showed more potent anti-tumor activity in CAR T-cells than deleting PD-1 alone [102]. As a negative regulator of T-cell persistence, LAG-3 contends with CD4 for binding MHC-II while suppressing T-cell function [103, 104]. CRISPR can be used to generate LAG-3 knockout CAR T cells with strengthened T-cell functionality [93]. Applying CRISPR/Cas9 to knockout diacylglycerol kinase (DGK) facilitates CD3 signaling and renders CAR-T cells resistant to immunosuppressive factors, such as TGF-$\beta$ and prostaglandin E2 [105].

Outside of immune checkpoints, CRISPR can be used to inhibit certain metabolic regulators, transcriptional factors, and signaling molecules that contribute to T-cell-intrinsic resistance. Due to Fas-FasL-dependent activation-induced cell death (AICD) attenuating CAR-T cell activity, the Fas ligand is another candidate target for CRISPR knockout [106]. A triple knockout of endogenous TCR, HLA-I, and FAS allows Fas-resistant universal CAR T-cells to display prolonger persistence in vitro and in vivo. Given the pleiotropic roles of TGF-$\beta$, Tang et al. utilized CRISPR to knockout TGF-$\beta$ receptor (TGFBR2) in CAR-T cells, rendering them unresponsive to exogenous TGF-$\beta$ signaling while exhibiting better tumor elimination efficacy in vivo [107]. Additionally, inhibition of NR4A transcriptional factor using CRISPR/Cas9 results in the downregulation of PD1 and TIM-3 [108]. Therefore, the potential can be seen for CRISPR/Cas9 to ameliorate “immune brakes” and develop the next generation of ACT.

### Reducing “on-target, off-tumor” toxicity

The “on-target, off-tumor” toxicity often occurs wherein CAR T cells cannot distinguish tumor and healthy cells expressing the same antigen. Cytokine release syndrome (CRS) is of widespread concern by triggering a positive feedback loop: activated immune cells release excessive cytokines that boost more immune cell activation, which would be fatal if not immediately managed.

**Fig. 6:**
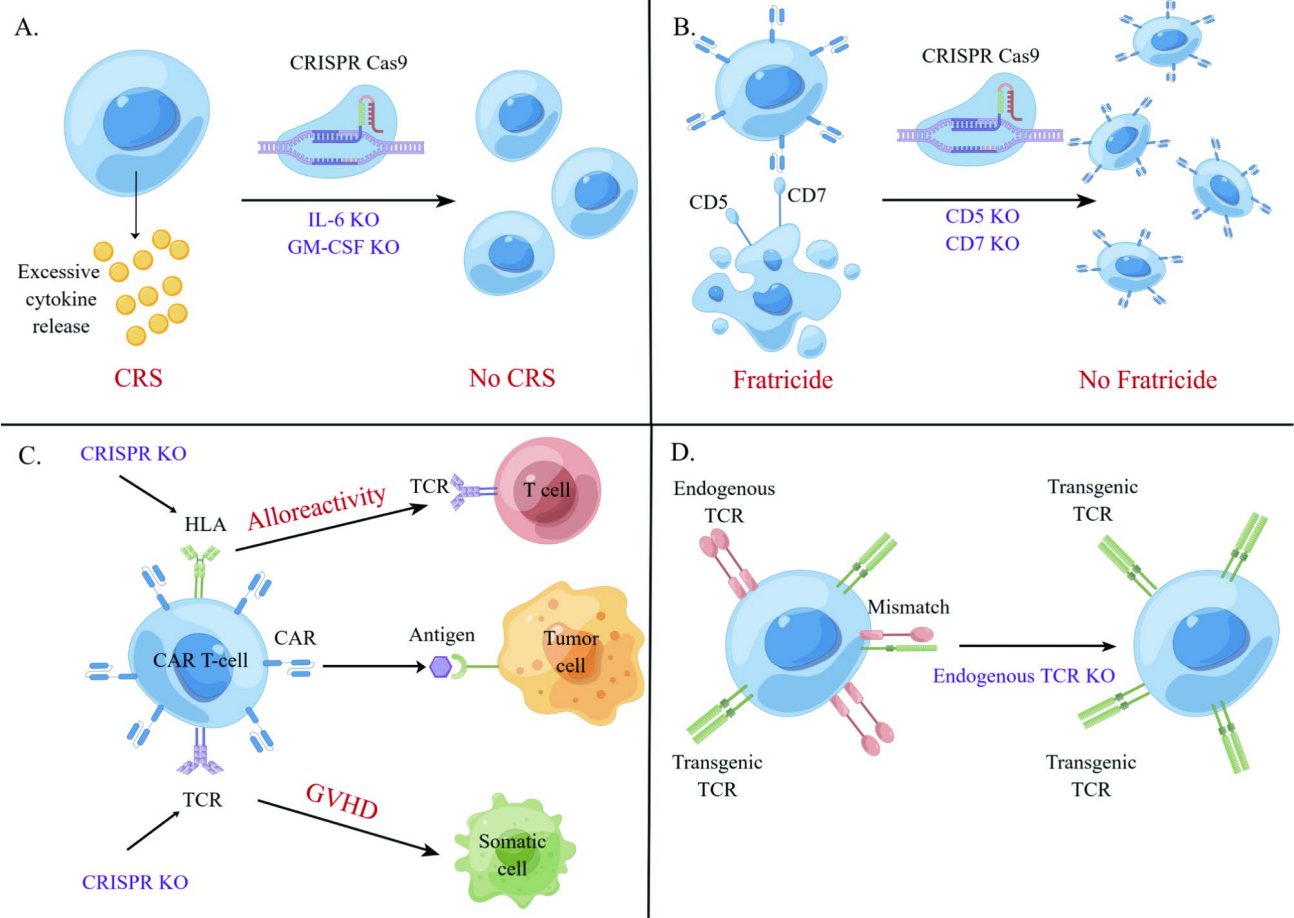
Innovations of CRISPR to improve the safety and scalability of ACT. **A**. CRISPR/Cas9-mediated cytokine knockout could avoid cytokine release syndrome (CRS). **B**. CRISPR/Cas9 could be used to knock out target antigens in off-target cells, such as CD7. **C**. CRISPR/Cas9 could be used to generate universal CAR T-cells via deleting endogenous TCR and HLA, reducing the risk of Graft-versus-host disease (GVHD) and alloreactivity. **D**. CRISPR/Cas9 could be used to generate universal TCR T-cells via knocking out endogenous TCR, overcoming on-target off-tumor challenges to broaden disease applications. Abbreviations: CRS, cytokine release syndrome; GVHD, Graft-versus-host disease; TCR, T cell receptor; KO, knock out

CRISPR/Cas9-mediated cytokine modulation has the potential to reduce “on-target, off-tumor” toxicity **(**Fig. 6A**)**. Firstly, CRISPR/Cas9 knock-in technology permits engineered T cells to express bi-allelic or sequential genes in a site-specific manner [109, 110]. More specifically, cytokine-encoding cassettes could be knocked in specific gene loci via CRISPR/Cas9, placing cytokine expression under the control of endogenous promoters. Applying this strategy, IL-15 is inserted into the IL-13 gene locus, controlling IL-15 secretion under the IL-13 promoter in a T-cell activation-dependent manner [111]. Another strategy is the CRISPR/Cas9 knock-out of genes encoding cytokines that drive CRS. CRISPR/Cas9 disruption of GM-CSF in CAR T cells could minimize risks of CRS and neuroinflammation while not compromising anti-tumor activity [112]. Likewise, as one of the most important initiators to amplify cytokine release, IL-6 knockdown can potentially ameliorate CRS and improve the safety of CAR T cell therapy [113].

Alternatively, CRISPR/Cas9 could knock out specific antigens on off-target cells. Since CD33 is widely distributed in normal and malignant cells in acute myeloid leukemia (AML), targeting it leads to serious myeloablation in treated patients. To address this limitation, a synthetic strategy that combines CD33-targeted CAR T-cell therapy with CD33-knockout hematopoietic stem cell transplantation creates attractive on-target efficacy and reduced off-tumor toxicity [114–116].

Fratricide is another on-target off-tumor challenge when treating T-cell malignancies. Co-expression of candidate antigens by CAR T-cells and malignant T cells, such as CD7 and CD5, gives rise to self-activation and self-killing during CAR T-cell manufacture. CRISPR/Cas9 knockout can overcome this barrier by disrupting the expression of surface antigens in T cells prior to CAR transduction **(**Fig. 6B**)**. Fratricide-resistant CAR T cells have been successfully produced by targeting CD7 [117, 118] and CD5 [119]. Overall, CRISPR could ameliorate “on-target, off-tumor” toxicity to improve the safety of ACT.

### Generating “off-the-shelf” allogeneic T cells

The clinical benefit of ACT is greatly blocked by the costly, time-consuming, low-yield manufacturing of autologous T-cells from patients’ own. Especially patients with lymphocyte depletion often have no time to wait for autologous T-cell production before rapid tumor progression [120]. This issue could be circumvented by creating “off-the-shelf” allogeneic T-cell with high cost-effectiveness, scalability, and standardized production [121].

#### Universal CAR T cells

Due to the presence of endogenous TCR and HLA on donor T cells, universal CAR T-cells often meet the challenges of Graft-Versus-Host-Disease (GVHD) and alloreactivity (host-versus-graft response) [122]. Recent CRISPR/Cas9 maturation can overcome these barriers by silencing the surface expression of TCR and HLA on T-cells. TCR could be silenced by CRISPR/Cas9 knockout of the TCR$\alpha$ subunit constant (TRAC) or TCR$\beta$ gene (TCRB), diminishing the occurrence of GVHD [91]. Additionally, $\beta$2 macroglobulin (B2M) forms heterodimers with HLA-I, necessary for HLA-I surface expression [123]. The therapeutic potential of the CRISPR system has been demonstrated in generating allogeneic CD19-directed CAR T cells with a double knockout of TCR and B2M, reducing the risk of autoimmunity [124]. By applying multiplex CRISPR, Liu et al. generated CAR-T cells with triple knockout genes (TRAC, B2M, and PD-1), which produced more cytokine release than standard CD19 CAR and double knockout while not compromising their potent efficacy [91].

Further success has been witnessed in a targeted knock-in of the CAR into the TRAC locus via HDR CRISPR/Cas9 mechanism while synchronously knocking out the native TCR to produce universal T cells. Besides, conventional CAR T-cells that employ lentiviral vectors for transduction and integration of CARs into T-cells usually bring risks of random insertions [125]. Thereby, placing CAR under the control of endogenous TRAC promoter will drive stable receptor expression. Instead of disrupting TRAC directly, studies directed a CD19-specific CAR to the TRAC locus, which not only silence endogenous TCR but also leads to uniform CAR expression [109, 126]. More specifically, a guide RNA was designed to target the 5’ end of the first exon of TRAC, leading to the silence of TRAC that encodes the native TCR, while an adeno-associated virus (AAV) vector was applied to transduce CAR expression to a defined location in the genome. The resulting universal T cells show less insertional carcinogenesis, reduced tonic signaling, delayed T-cell exhaustion, and enhanced tumor elimination [109]. Overall, the TRAC locus is an ideal target for CAR knock-in and TCR knock-out to prepare universal allogeneic T cells, eliminating concerns of insertional carcinogenesis and TCR-induced autoimmunity [109, 110] **(**Fig. 6C**)**.

#### Universal TCR modified T cells

CRISPR/Cas9 also spurred an interest in universal allogeneic TCR-modified T-cell therapy, another sunset of ACT with broader applicability than CAR T cells. T cells are isolated from donors’ blood, genetically modified to encode the TCR protein, and then expanded and infused into patients [127]. TCRs are potential cancer immunotherapy candidates, expressed as either $\alpha \beta$ or $\gamma \delta$ heterodimers, which can be precisely knocked into specific genetic loci by CRISPR/Cas9. A high frequency of transgenic TCR is necessary to enhance TCR T-cell therapy. However, one major defect is the endogenous TCR competing with transduced TCR to unify with CD3 [128]. Secondly, mismatches between endogenous and transduced TCR will create four distinct TCR dimers with unpredictable epitope specificities leading to dangerous autoimmunity or GVHD [129].

Abrogation of endogenous TCR is a promising strategy to address TCR competition and mismatches. Knockout of endogenous TCR$\alpha \beta$ has been achieved by CRISPR/Cas9 editing of the TRAC or TCRB loci. Legut et al. demonstrated that endogenous TCR$\beta$ knockout efficiently improves the expression of transgenic $\alpha \beta$ and$\gamma \delta$ TCRs, boosting more potent antitumor responses while not aggravating T-cell exhaustion [129]. This finding can be attributed to the low affinity of endogenous TCR$\alpha$ to dimerize with other TCR subunits. Since TCR mispairing remains prevalent following TRAC silencing, simultaneous TRAC and TCRB knockout in single transduction via CRIPSR/Cas9 RNPs allows for increased transgenic TCR expression in edited T cell populations [130]. Hence, to construct CRISPR-mediated TCR abrogation, sgRNAs are designed to target the first exon of TRAC or TCRB, followed by T-cell transduction with lentiviral particles **(**Fig. 6D**)**.

## The next-generation of CRISPR/Cas9-modified adoptive cell therapy

Cancer immunotherapy engineered by CRISPR/Cas9 system is making continuous progress for clinical use, with various clinical trials now underway **(**Table-3**)**. Alternative strategies have started to emerge with improved efficiency and lower off-target effects compared to conventional CRISPR-Cas9. Future perspectives of CRISPR/Cas9-based methodology could improve its bioavailability and therapeutic potential [131]. The next-generation gene editing technologies that change the sequence of a single base pair at a target site without introducing DSBs offer a platform to bring unprecedented precision and improve the safety of CRISPR-engineered ACT via reducing off-target mutations. The two latest innovations include base editors and prime editors.

**Table 3:** Clinical trials of adoptive cell therapy using CRISPR/Cas9 technology

Identifier	Phase	Target	Cancer type	Immunotherapy	Status	Last update
NCT04637763	Phase 1	CD19	B-cell non-Hodgkin lymphoma	Allogeneic CRISPR/Cas9-engineered T cells (CB-010)	Recruiting	2023/6/15
NCT05643742	Phase 1/2	CD19	B cell malignancies	Allogeneic CRISPR/Cas9-engineered T cells (CTX112)	Recruiting	2023/3/31
NCT05662904	Phase 1	CD33	AML	Allogeneic hematopoietic stem cell transplantation	Not yet recruiting	2022/12/23
NCT05795595	Phase 1/2	CD70	Solid tumors	Allogeneic CD70-directed CAR-T cell (CTX131)	Recruiting	2023/4/19
NCT04502446	Phase 1	CD70	T or B cell malignancies	Allogeneic CRISPR/Cas9-engineered T cells (CTX130)	Active	2023/4/27
NCT04438038	Phase 1	CD70	Renal cell carcinoma	Allogeneic CRISPR/Cas9-engineered T cells (CTX130)	Active	2023/5/11
NCT04244656	Phase 1	BCMA	Multiple myeloma	Allogeneic CRISPR/Cas9-engineered T cells (CTX120)	Active	2022/7/18
NCT04426669	Phase 1/2	CISH	Gastro-intestinal (GI) cancer	Genetically engineered TIL	Recruiting	2023/3/3
NCT05566223	Phase 1/2	CISH	Non-small cell lung cancer	Genetically engineered TIL	Not yet recruiting	2022/12/9
NCT04035434	Phase 1/2	Endo-TCR	B cell malignancies	Allogeneic CRISPR/Cas9-engineered T cells (CTX110)	Recruiting	2023/7/17
NCT03166878	Phase 1/2	Endo-TCR/B2M	B-cell leukemia and lymphoma	UCART019	Unknown	2017/6/23
NCT03545815	Phase 1	Endo-TCR/PD-1	Solid tumors	Anti-mesothelin CRISPR/Cas9-mediated CAR-T cell	Unknown	2020/8/10
NCT03399448	Phase 1	Endo-TCR/PD-1	Multiple myeloma; Melanoma; Synovial sarcoma	NY-ESO-1 TCR-T	Terminated	2023/6/22
NCT03690011	Phase 1	CD7	T-cell malignancies	Autologous T cells	Recruiting	2023/4/28
NCT04264078	Phase 1	CD7/TRAC	T/NK cell hematologic malignancies	Allogeneic CAR-T cell	Unknown	2021/6/28
NCT04557436	Phase 1	CD52/TRAC	B-ALL	Allogeneic CAR-T cell	Active	2023/5/31
NCT03747965	Phase 1	PD-1	Solid tumors	Mesothelin-directed CAR-T cell	Unknown	2018/11/20
NCT04417764	Phase 1	PD-1	Hepatocellular carcinoma	Engineered T cells	Recruiting	2023/2/9
NCT02793856	Phase 1	PD-1	Non-small cell lung cancer	Engineered T cells	Completed	2021/1/12
NCT03081715	Phase 1	PD-1	Esophageal cancer	Engineered T cells	Completed	2019/6/12

**Abbreviations.** CAR, chimeric antigen receptor; TIL, Tumor infiltrating lymphocytes; ALL, acute lymphocytic leukemia; AML, acute myeloid leukemia

Base editors, consisting of a catalytically deficient Cas9 variant and a deaminase, induce specific point mutations without creating DSBs or depending on HDR [132–134]. Two major types of base editors, including cytosine base editor (CBE) and adenine base editor (ABE), mediate four possible transversions: C to T, T to C, A to G, and G to A [132, 135, 136]. A prominent advantage of base editors is that genetic modification occurs with single-nucleotide-level precision, minimizing off-target effects while maintaining efficient on-target response. This technology has been validated in multiplexed gene disruption in primary T cells, highlighting its great prospects in the context of ACT [137]. However, base editors cannot avoid single-nucleotide variants (SNV) and even act on RNA, limiting its genome editing specificity [138].

The prime editor consists of a nCas9 and a reverse transcriptase. The modified gRNA guides a site-specific binding while serving as a template to synthesize new DNA strands. Prime editing with no restriction of editing window is more versatile than base editing to mediate all possible base transitions [139]. Meanwhile, prime editing displays high specificity with little off-target effects than conventional CRISPR/Cas9. Moreover, this approach leads to fewer indels than genetic modification via CRISPR-mediated HDR [140, 141]. Efforts to apply prime editing in human cell lines have yielded pleasant success. More studies are still required to exploit this technology in immune cells and fully characterize its safety profile.

## Challenges and perspectives

The fast-developing CRISPR/Cas9 has revolutionized cancer immunotherapy by specifically modifying immune cells, but it still has many limitations and risks. Of note, major challenges include off-targeting toxicity and mutations, Cas9-related immunogenicity, delivery method, T-cell exhaustion and senescence, and immunosuppressive factors in the TME. The balance between efficacy and toxicity will determine the scalability of ACT in clinical use.

The safety of the CRISPR/Cas9 system in cancer immunotherapy is always the priority. Firstly, off-targeting is a significant risk factor of CRISPR/Cas9. When applying the CRISPR system to a complicated genomic species, DSB may occur at off-target sites owing to the similarity within the genome, potentially leading to unintended mutations [142]. Therefore, reducing the off-target activity of Cas9 can be achieved by developing Cas9 variants, designing sgRNA with high specificity, choosing a suitable delivery system, and targeting sequences with low GC content [143–145]. Secondly, the human immune system may recognize Cas9 as a foreign protein and develop autoimmune responses, manifesting the potential risk of inflammation and even mortality during CRISPR/Cas9-based cancer therapy [146]. Several strategies have been proposed to overcome immunogenicity: (1) co-administrating immunosuppressants, (2) modifying the secondary structure of sgRNA, and (3) targeting immune-privileged organs [146–148]. Moreover, NHEJ and HDR may generate deleterious DSB repair byproducts that threaten genome stability during CRISPR genome editing [149, 150]. PEM-sequencing, HTGHT, and SuperQ emerged to help distinguish various DNA repair products [151]. Additionally, the safe delivery of the CRISPR system to the target site is another concern. Compared to viral delivery vectors, non-viral delivery vectors allow for ACT with a lower risk of immunogenicity, greater capacity, and more precise targeting [152, 153]. Nanotechnology-based delivery of CRISPR/Cas9 recently opened a new way for clinical translation, as reviewed elsewhere [154]. CRISPR delivery strategy will be continuously optimized to become an effective therapeutic tool. Other challenges of CRISPR/Cas9 therapeutic use, involving low-efficacy delivery of gene editing components, inefficient repair by DSBs via HDR, and high-frequency genomic mutations at target sites, also require better solutions in the future [155].

Importantly, there has been a rapid expansion of variant Cas proteins that broaden the applicability of the CRISPR toolbox as a research or therapeutic tool. One source of variants arises from the Cas9 orthologues that possess distinct characteristics. For instance, the *Staphylococcus aureus* Cas9 (SaCas9) identifies a different PAM sequence from SpCas9, thereby providing an avenue to target alternative genomic loci. Some enzymes, including SaCas9, *Neisseria meningitidis* Cas9 (NmeCas9), and *Campylobacter jejuni* Cas9 (CjCas9), exhibit smaller sizes than SpCas9. This feature facilitates their much easier incorporation into delivery vectors with size restrictions, such as AAV [156–158]. Also, Cas9 variants have been developed to address the limitations of DSB cleavage. Specifically, Cas9 nickase (Cas9n) variants have been modified in the RuvC or HNH domain, allowing Cas9 to exclusively cleave only the targeted or non-targeted DNA strand, respectively [144]. Mutations occur in both catalytic domains to produce the dCas9 variant. Thereby, dCas9 is independent of Cas9 catalytic activity and can be easily attached to functional enzymes, creating diverse site-specific modifications [159]. Furthermore, other families of Cas proteins are also utilized in genome editing. Cas12 (known as Cpf1) only requires crRNA guidance for DNA targeting, streamlining the procedure of target multiplexing [160]. Cas13 cleaves RNA specifically instead of DNA, creating an alternative approach to modulating gene expression [161]. Given the continuous identification of naturally occurring Cas proteins and the popularization of engineered Cas proteins, the expanded repertoire of CRISPR tools allows for selecting an appropriate platform to maximize therapeutic potential.

Of note, CRISPR/Cas9-mediated editing approach offers a promising means for precision or personalized medicine. Next-generation sequencing (NGS) technology allows for the availability to cancer genomic profiles, which could be used as a base by CRISPR/Cas9 for correcting the mutated genes [162, 163]. Therefore, the combination of CRISPR/Cas9 system with NGS technology holds the promise to speed up the identification and targeting of tumor-driven or synergistic lethal genes, helping to improve cancer immunotherapy choices.

## Conclusion

Given the advantages of mouse models and organoids that fully characterize the TME, CRISPR screening provides a powerful genetic editing platform to discover important tumor-associated targets and monitor immunotherapy regimens. Despite the advent of ACT shedding light on tumor treatment, its wide application remains limited due to some hurdles. The versatile CRISPR/Cas9 system is set to surmount these barriers to improve ACT’s potency, scalability, and safety. Trials for novel CRISPR-engineered ACT are still in early infancy, and preliminary trial results will lay the foundation for the clinical use of CRISPR-modified immunotherapy. Using CRISPR/Cas9 to understand cellular and molecular mechanisms underpinning tumor-immune interactions will provide a solid rationale to engineer ACT to achieve its full therapeutic potential.

## Data Availability

Not applicable.

## Declarations

**Ethic approval and consent to participate**.

## Abbreviations

CRISPR: clustered regulatory interspaced short palindromic repeats
Cas9: CRISPR-associated protein 9
ZFNs: Zine finger nucleases
TALENs: transcription activator-like effector nucleases
sgRNA: single guide RNA
DSB: double-strand break
NHEJ: non-homologous end joining
HDR: homology directed repair
TME: tumor microenvironment
ACT: adoptive cell therapy
CAR: chimeric antigen receptor
TCR: T cell receptor
TIL: tumor-infiltrating lymphocytes
SpCas9: *Streptococcus pyogenes* Cas9
crRNA: CRISPR-derived RNA
tracrRNA: transactivating crRNA
RNP: ribonucleoprotein
PAM: protospacer adjacent motif
dCas9: catalytically dead Cas9
CRISPRa: CRISPR activation
CRISPRi: CRISPR interference
ICB: immune checkpoint blockade
CRISPR-GEMMs: CRISPR-mediated genetically engineered mouse models
LCMV: lymphocytic choriomeningitis
T _FH_: T follicular helper cell
OVA: ovalbumin
PDOs: patient-derived organoids
PDCs: patient-derived cancer cells
PDXs: patient-derived xenografts
iPSCs: induced pluripotent stem cells
SSCs: somatic stem cells
MMR: mismatch repair
SCLC: small cell lung cancer
RNAi: RNA interference
TKIs: tyrosine kinase inhibitors
T_reg_: Regulatory T cell
FACS: fluorescence-activated cell sorting
APC: ntigen-presenting cell
PDAC: pancreatic ductal adenocarcinoma
MDSC: myeloid-derived suppressor cell
TF: transcriptional factor
DGK: diacylglycerol kinase
AICD: activation-induced cell death
TGFBR2: TGF-$\beta$ receptor
CRS: cytokine release syndrome
GVHD: Graft-Versus-Host-Disease
TRAC: TCR$\alpha$ subunit constant
TCRB: TCR$\beta$ gene
B2M: $$\beta$$2 macroglobulin
AAV: adeno-associated virus
CBE: cytosine base editor
ABE: adenine base editor
SNV: single-nucleotide variants
SaCas9: *Staphylococcus aureus* Cas9
NmeCas9: *Neisseria meningitidis* Cas9
CjCas9: *Campylobacter jejuni* Cas9
Cas9n: Cas9 nickase
NGS: next-generation sequencing

## References

[1] Ishino Y, Shinagawa H, Makino K, Amemura M, Nakata A (1987). Nucleotide sequence of the iap gene, responsible for alkaline phosphatase isozyme conversion in Escherichia coli, and identification of the gene product. J Bacteriol.

[2] Cui Z, Liu H, Zhang H, Huang Z, Tian R, Li L (2021). The comparison of ZFNs, TALENs, and SpCas9 by GUIDE-seq in HPV-targeted gene therapy. Mol Therapy Nucleic Acids.

[3] Gaj T, Gersbach CA, Barbas CF (2013). 3rd. ZFN, TALEN, and CRISPR/Cas-based methods for genome engineering. Trends Biotechnol.

[4] Lino CA, Harper JC, Carney JP, Timlin JA (2018). Delivering CRISPR: a review of the challenges and approaches. Drug Delivery.

[5] Hsu PD, Lander ES, Zhang F (2014). Development and applications of CRISPR-Cas9 for genome engineering. Cell.

[6] Doudna JA, Charpentier E (2014). Genome editing. The new frontier of genome engineering with CRISPR-Cas9. Sci (New York NY).

[7] Martinez-Lage M, Puig-Serra P, Menendez P, Torres-Ruiz R, Rodriguez-Perales S. CRISPR/Cas9 for Cancer Therapy: hopes and challenges. Biomedicines. 2018;6(4). 10.3390/biomedicines6040105 PMC6315587 30424477

[8] Zhang JH, Adikaram P, Pandey M, Genis A, Simonds WF (2016). Optimization of genome editing through CRISPR-Cas9 engineering. Bioengineered.

[9] Cho SW, Kim S, Kim JM, Kim JS (2013). Targeted genome engineering in human cells with the Cas9 RNA-guided endonuclease. Nat Biotechnol.

[10] Cong L, Ran FA, Cox D, Lin S, Barretto R, Habib N (2013). Multiplex genome engineering using CRISPR/Cas systems. Sci (New York NY).

[11] Jinek M, East A, Cheng A, Lin S, Ma E, Doudna J (2013). RNA-programmed genome editing in human cells. eLife.

[12] Mali P, Yang L, Esvelt KM, Aach J, Guell M, DiCarlo JE et al. RNA-guided human genome engineering via Cas9. Science (New York, NY). 2013;339(6121):823–6. 10.1126/science.1232033 PMC3712628 23287722

[13] Lim WA, June CH (2017). The principles of Engineering Immune cells to treat Cancer. Cell.

[14] Jindal V, Arora E, Gupta S (2018). Challenges and prospects of chimeric antigen receptor T cell therapy in solid tumors. Med Oncol (Northwood Lond Engl).

[15] Pourcel C, Salvignol G, Vergnaud G (2005). CRISPR elements in Yersinia pestis acquire new repeats by preferential uptake of bacteriophage DNA, and provide additional tools for evolutionary studies. Microbiology.

[16] van der Oost J, Westra ER, Jackson RN, Wiedenheft B (2014). Unravelling the structural and mechanistic basis of CRISPR-Cas systems. Nat Rev Microbiol.

[17] Barrangou R, Fremaux C, Deveau H, Richards M, Boyaval P, Moineau S (2007). CRISPR provides acquired resistance against viruses in prokaryotes. Sci (New York NY).

[18] Jinek M, Chylinski K, Fonfara I, Hauer M, Doudna JA, Charpentier E (2012). A programmable dual-RNA-guided DNA endonuclease in adaptive bacterial immunity. Sci (New York NY).

[19] Sapranauskas R, Gasiunas G, Fremaux C, Barrangou R, Horvath P, Siksnys V (2011). The Streptococcus thermophilus CRISPR/Cas system provides immunity in Escherichia coli. Nucleic Acids Res.

[20] Sternberg SH, Redding S, Jinek M, Greene EC, Doudna JA (2014). DNA interrogation by the CRISPR RNA-guided endonuclease Cas9. Nature.

[21] Makarova KS, Grishin NV, Shabalina SA, Wolf YI, Koonin EV (2006). A putative RNA-interference-based immune system in prokaryotes: computational analysis of the predicted enzymatic machinery, functional analogies with eukaryotic RNAi, and hypothetical mechanisms of action. Biol Direct.

[22] Gasiunas G, Barrangou R, Horvath P, Siksnys V (2012). Cas9-crRNA ribonucleoprotein complex mediates specific DNA cleavage for adaptive immunity in bacteria. Proc Natl Acad Sci USA.

[23] Anders C, Niewoehner O, Duerst A, Jinek M (2014). Structural basis of PAM-dependent target DNA recognition by the Cas9 endonuclease. Nature.

[24] Lieber MR, Ma Y, Pannicke U, Schwarz K (2003). Mechanism and regulation of human non-homologous DNA end-joining. Nat Rev Mol Cell Biol.

[25] Rouet P, Smih F, Jasin M (1994). Introduction of double-strand breaks into the genome of mouse cells by expression of a rare-cutting endonuclease. Mol Cell Biol.

[26] Cromie GA, Connelly JC, Leach DR (2001). Recombination at double-strand breaks and DNA ends: conserved mechanisms from phage to humans. Mol Cell.

[27] He C, Han S, Chang Y, Wu M, Zhao Y, Chen C (2021). CRISPR screen in cancer: status quo and future perspectives. Am J cancer Res.

[28] Platt RJ, Chen S, Zhou Y, Yim MJ, Swiech L, Kempton HR (2014). CRISPR-Cas9 knockin mice for genome editing and cancer modeling. Cell.

[29] Chu VT, Weber T, Graf R, Sommermann T, Petsch K, Sack U (2016). Efficient generation of Rosa26 knock-in mice using CRISPR/Cas9 in C57BL/6 zygotes. BMC Biotechnol.

[30] Li R, Xia X, Wang X, Sun X, Dai Z, Huo D (2020). Generation and validation of versatile inducible CRISPRi embryonic stem cell and mouse model. PLoS Biol.

[31] Shi S, Gu S, Han T, Zhang W, Huang L, Li Z (2020). Inhibition of MAN2A1 enhances the Immune response to Anti-PD-L1 in human tumors. Clin cancer Research: Official J Am Association Cancer Res.

[32] Manguso RT, Pope HW, Zimmer MD, Brown FD, Yates KB, Miller BC (2017). In vivo CRISPR screening identifies Ptpn2 as a cancer immunotherapy target. Nature.

[33] Lawson KA, Sousa CM, Zhang X, Kim E, Akthar R, Caumanns JJ (2020). Functional genomic landscape of cancer-intrinsic evasion of killing by T cells. Nature.

[34] Dubrot J, Du PP, Lane-Reticker SK, Kessler EA, Muscato AJ, Mehta A (2022). In vivo CRISPR screens reveal the landscape of immune evasion pathways across cancer. Nat Immunol.

[35] Li F, Huang Q, Luster TA, Hu H, Zhang H, Ng WL (2020). In vivo epigenetic CRISPR screen identifies Asf1a as an immunotherapeutic target in Kras-Mutant Lung Adenocarcinoma. Cancer Discov.

[36] Wang X, Tokheim C, Gu SS, Wang B, Tang Q, Li Y (2021). In vivo CRISPR screens identify the E3 ligase Cop1 as a modulator of macrophage infiltration and cancer immunotherapy target. Cell.

[37] Wang G, Chow RD, Zhu L, Bai Z, Ye L, Zhang F (2020). CRISPR-GEMM pooled mutagenic screening identifies KMT2D as a major modulator of Immune Checkpoint Blockade. Cancer Discov.

[38] Chow RD, Guzman CD, Wang G, Schmidt F, Youngblood MW, Ye L (2017). AAV-mediated direct in vivo CRISPR screen identifies functional suppressors in glioblastoma. Nat Neurosci.

[39] Kamber RA, Nishiga Y, Morton B, Banuelos AM, Barkal AA, Vences-Catalán F (2021). Inter-cellular CRISPR screens reveal regulators of cancer cell phagocytosis. Nature.

[40] Chapman NM, Chi H (2022). Metabolic adaptation of lymphocytes in immunity and Disease. Immunity.

[41] Fu G, Guy CS, Chapman NM, Palacios G, Wei J, Zhou P (2021). Metabolic control of T(FH) cells and humoral immunity by phosphatidylethanolamine. Nature.

[42] Del Sutra A, Menegatti S, Fuentealba J, Lucibello F, Perrin L, Helft J (2021). In vivo genome-wide CRISPR screens identify SOCS1 as intrinsic checkpoint of CD4(+) T(H)1 cell response. Sci Immunol.

[43] Sugiura A, Andrejeva G, Voss K, Heintzman DR, Xu X, Madden MZ (2022). MTHFD2 is a metabolic checkpoint controlling effector and regulatory T cell fate and function. Immunity.

[44] Dong MB, Wang G, Chow RD, Ye L, Zhu L, Dai X (2019). Systematic Immunotherapy Target Discovery using genome-scale in vivo CRISPR screens in CD8 T cells. Cell.

[45] Ye L, Park JJ, Dong MB, Yang Q, Chow RD, Peng L (2019). In vivo CRISPR screening in CD8 T cells with AAV-Sleeping Beauty hybrid vectors identifies membrane targets for improving immunotherapy for glioblastoma. Nat Biotechnol.

[46] Baliou S, Adamaki M, Kyriakopoulos AM, Spandidos DA, Panayiotidis M, Christodoulou I (2018). CRISPR therapeutic tools for complex genetic disorders and cancer (review). Int J Oncol.

[47] Lo YH, Kolahi KS, Du Y, Chang CY, Krokhotin A, Nair A (2021). A CRISPR/Cas9-Engineered ARID1A-Deficient human gastric Cancer Organoid Model reveals essential and nonessential modes of Oncogenic Transformation. Cancer Discov.

[48] Drost J, van Jaarsveld RH, Ponsioen B, Zimberlin C, van Boxtel R, Buijs A (2015). Sequential cancer mutations in cultured human intestinal stem cells. Nature.

[49] Matano M, Date S, Shimokawa M, Takano A, Fujii M, Ohta Y (2015). Modeling Colorectal cancer using CRISPR-Cas9-mediated engineering of human intestinal organoids. Nat Med.

[50] Schwank G, Koo BK, Sasselli V, Dekkers JF, Heo I, Demircan T (2013). Functional repair of CFTR by CRISPR/Cas9 in intestinal stem cell organoids of cystic fibrosis patients. Cell Stem Cell.

[51] Dekkers JF, Whittle JR, Vaillant F, Chen HR, Dawson C, Liu K (2020). Modeling Breast Cancer using CRISPR-Cas9-Mediated Engineering of human breast organoids. J Natl Cancer Inst.

[52] Ng SR, Rideout WM, Akama-Garren EH, Bhutkar A, Mercer KL, Schenkel JM (2020). CRISPR-mediated modeling and functional validation of candidate Tumor suppressor genes in small cell Lung cancer. Proc Natl Acad Sci USA.

[53] Shalem O, Sanjana NE, Zhang F (2015). High-throughput functional genomics using CRISPR-Cas9. Nat Rev Genet.

[54] Doench JG (2018). Am I ready for CRISPR? A user’s guide to genetic screens. Nat Rev Genet.

[55] Strutt SC, Torrez RM, Kaya E, Negrete OA, Doudna JA. RNA-dependent RNA targeting by CRISPR-Cas9. eLife. 2018;7. 10.7554/eLife.32724 PMC5796797 29303478

[56] Bock C, Datlinger P, Chardon F, Coelho MA, Dong MB, Lawson KA et al. High-content CRISPR screening. Nat Reviews Methods Primers. 2022;2(1). 10.1038/s43586-022-00098-7 PMC10200264 37214176

[57] Kalbasi A, Ribas A (2020). Tumour-intrinsic resistance to immune checkpoint blockade. Nat Rev Immunol.

[58] Wang T, Wei JJ, Sabatini DM, Lander ES. Genetic screens in human cells using the CRISPR-Cas9 system. Science (New York, NY). 2014;343(6166):80 – 4. 10.1126/science.1246981 PMC3972032 24336569

[59] Krall EB, Wang B, Munoz DM, Ilic N, Raghavan S, Niederst MJ et al. KEAP1 loss modulates sensitivity to kinase targeted therapy in Lung cancer. eLife. 2017;6. 10.7554/eLife.18970 PMC5305212 28145866

[60] Crompton JG, Sukumar M, Restifo NP (2014). Uncoupling T-cell expansion from effector differentiation in cell-based immunotherapy. Immunol Rev.

[61] Zhou Y, Zhu S, Cai C, Yuan P, Li C, Huang Y (2014). High-throughput screening of a CRISPR/Cas9 library for functional genomics in human cells. Nature.

[62] Rosenberg SA, Restifo NP (2015). Adoptive cell transfer as personalized immunotherapy for human cancer. Sci (New York NY).

[63] Wei J, Long L, Zheng W, Dhungana Y, Lim SA, Guy C (2019). Targeting REGNASE-1 programs long-lived effector T cells for cancer therapy. Nature.

[64] Kumar S, Zeng Z, Bagati A, Tay RE, Sanz LA, Hartono SR (2021). CARM1 inhibition enables immunotherapy of resistant tumors by Dual Action on Tumor Cells and T cells. Cancer Discov.

[65] Chen Z, Arai E, Khan O, Zhang Z, Ngiow SF, He Y (2021). In vivo CD8(+) T cell CRISPR screening reveals control by Fli1 in Infection and cancer. Cell.

[66] Belk JA, Yao W, Ly N, Freitas KA, Chen YT, Shi Q (2022). Genome-wide CRISPR screens of T cell exhaustion identify chromatin remodeling factors that limit T cell persistence. Cancer Cell.

[67] Shang W, Jiang Y, Boettcher M, Ding K, Mollenauer M, Liu Z (2018). Genome-wide CRISPR screen identifies FAM49B as a key regulator of actin dynamics and T cell activation. Proc Natl Acad Sci USA.

[68] Schumann K, Raju SS, Lauber M, Kolb S, Shifrut E, Cortez JT (2020). Functional CRISPR dissection of gene networks controlling human regulatory T cell identity. Nat Immunol.

[69] Burr ML, Sparbier CE, Chan KL, Chan YC, Kersbergen A, Lam EYN (2019). An evolutionarily conserved function of polycomb silences the MHC Class I Antigen Presentation Pathway and enables Immune Evasion in Cancer. Cancer Cell.

[70] Burr ML, Sparbier CE, Chan YC, Williamson JC, Woods K, Beavis PA (2017). CMTM6 maintains the expression of PD-L1 and regulates anti-tumour immunity. Nature.

[71] Gu SS, Zhang W, Wang X, Jiang P, Traugh N, Li Z (2021). Therapeutically increasing MHC-I expression potentiates Immune Checkpoint Blockade. Cancer Discov.

[72] Barkal AA, Brewer RE, Markovic M, Kowarsky M, Barkal SA, Zaro BW (2019). CD24 signalling through macrophage Siglec-10 is a target for cancer immunotherapy. Nature.

[73] Wang J, Sun J, Liu LN, Flies DB, Nie X, Toki M (2019). Siglec-15 as an immune suppressor and potential target for normalization cancer immunotherapy. Nat Med.

[74] Brentjens RJ, Davila ML, Riviere I, Park J, Wang X, Cowell LG (2013). CD19-targeted T cells rapidly induce molecular remissions in adults with chemotherapy-refractory acute lymphoblastic Leukemia. Sci Transl Med.

[75] Huang D, Miller M, Ashok B, Jain S, Peppas NA (2020). CRISPR/Cas systems to overcome challenges in developing the next generation of T cells for cancer therapy. Adv Drug Deliv Rev.

[76] Maude SL, Frey N, Shaw PA, Aplenc R, Barrett DM, Bunin NJ (2014). Chimeric antigen receptor T cells for sustained remissions in Leukemia. N Engl J Med.

[77] Ghaffari S, Torabi-Rahvar M, Omidkhoda A, Ahmadbeigi N. Impact of various culture conditions on ex vivo expansion of polyclonal T cells for adoptive immunotherapy. APMIS: acta pathologica, microbiologica, et immunologica Scandinavica. 2019;127(12):737–45. 10.1111/apm.12981 31273832

[78] Song DG, Ye Q, Poussin M, Harms GM, Figini M, Powell DJ (2012). CD27 costimulation augments the survival and antitumor activity of redirected human T cells in vivo. Blood.

[79] Kawalekar OU, RS OC, Fraietta JA, Guo L, McGettigan SE, Posey AD (2016). Distinct signaling of Coreceptors regulates specific metabolism pathways and impacts Memory Development in CAR T cells. Immunity.

[80] Buchan SL, Rogel A, Al-Shamkhani A (2018). The immunobiology of CD27 and OX40 and their potential as targets for cancer immunotherapy. Blood.

[81] Guercio M, Orlando D, Di Cecca S, Sinibaldi M, Boffa I, Caruso S (2021). CD28.OX40 co-stimulatory combination is associated with long in vivo persistence and high activity of CAR.CD30 T-cells. Haematologica.

[82] Curran KJ, Seinstra BA, Nikhamin Y, Yeh R, Usachenko Y, van Leeuwen DG (2015). Enhancing antitumor efficacy of chimeric antigen receptor T cells through constitutive CD40L expression. Mol Therapy: J Am Soc Gene Therapy.

[83] Kuhn NF, Purdon TJ, van Leeuwen DG, Lopez AV, Curran KJ, Daniyan AF (2019). CD40 Ligand-Modified Chimeric Antigen Receptor T Cells Enhance Antitumor Function by eliciting an endogenous Antitumor response. Cancer Cell.

[84] Hollmann CA, Owens T, Nalbantoglu J, Hudson TJ, Sladek R (2006). Constitutive activation of extracellular signal-regulated kinase predisposes diffuse large B-cell Lymphoma cell lines to CD40-mediated cell death. Cancer Res.

[85] Chmielewski M, Kopecky C, Hombach AA, Abken H (2011). IL-12 release by engineered T cells expressing chimeric antigen receptors can effectively Muster an antigen-independent macrophage response on Tumor cells that have shut down Tumor antigen expression. Cancer Res.

[86] Teague RM, Sather BD, Sacks JA, Huang MZ, Dossett ML, Morimoto J (2006). Interleukin-15 rescues tolerant CD8 + T cells for use in adoptive immunotherapy of established tumors. Nat Med.

[87] Hu B, Ren J, Luo Y, Keith B, Young RM, Scholler J (2017). Augmentation of Antitumor immunity by human and mouse CAR T cells secreting IL-18. Cell Rep.

[88] Singer M, Wang C, Cong L, Marjanovic ND, Kowalczyk MS, Zhang H (2016). A distinct Gene Module for Dysfunction Uncoupled from Activation in Tumor-infiltrating T cells. Cell.

[89] Klepsch V, Pommermayr M, Humer D, Brigo N, Hermann-Kleiter N, Baier G (2020). Targeting the orphan nuclear receptor NR2F6 in T cells primes tumors for immune checkpoint therapy. Cell Communication and Signaling: CCS.

[90] Cheng J, Zhao L, Zhang Y, Qin Y, Guan Y, Zhang T (2019). Understanding the mechanisms of Resistance to CAR T-Cell Therapy in Malignancies. Front Oncol.

[91] Liu X, Zhang Y, Cheng C, Cheng AW, Zhang X, Li N (2017). CRISPR-Cas9-mediated multiplex gene editing in CAR-T cells. Cell Res.

[92] Ren J, Liu X, Fang C, Jiang S, June CH, Zhao Y (2017). Multiplex Genome Editing to Generate Universal CAR T Cells Resistant to PD1 inhibition. Clin cancer Research: Official J Am Association Cancer Res.

[93] Zhang Y, Zhang X, Cheng C, Mu W, Liu X, Li N (2017). CRISPR-Cas9 mediated LAG-3 disruption in CAR-T cells. Front Med.

[94] Zhang W, Shi L, Zhao Z, Du P, Ye X, Li D (2019). Disruption of CTLA-4 expression on peripheral blood CD8 + T cell enhances anti-tumor efficacy in Bladder cancer. Cancer Chemother Pharmacol.

[95] Rupp LJ, Schumann K, Roybal KT, Gate RE, Ye CJ, Lim WA (2017). CRISPR/Cas9-mediated PD-1 disruption enhances anti-tumor efficacy of human chimeric antigen receptor T cells. Sci Rep.

[96] Su S, Zou Z, Chen F, Ding N, Du J, Shao J (2017). CRISPR-Cas9-mediated disruption of PD-1 on human T cells for adoptive cellular therapies of EBV positive gastric cancer. Oncoimmunology.

[97] Guo X, Jiang H, Shi B, Zhou M, Zhang H, Shi Z (2018). Disruption of PD-1 enhanced the anti-tumor activity of Chimeric Antigen Receptor T Cells against Hepatocellular Carcinoma. Front Pharmacol.

[98] Hu B, Zou Y, Zhang L, Tang J, Niedermann G, Firat E (2019). Nucleofection with plasmid DNA for CRISPR/Cas9-Mediated inactivation of programmed cell death protein 1 in CD133-Specific CAR T cells. Hum Gene Ther.

[99] Hu W, Zi Z, Jin Y, Li G, Shao K, Cai Q (2019). CRISPR/Cas9-mediated PD-1 disruption enhances human mesothelin-targeted CAR T cell effector functions. Cancer Immunol Immunotherapy: CII.

[100] Lu Y, Xue J, Deng T, Zhou X, Yu K, Deng L (2020). Safety and feasibility of CRISPR-edited T cells in patients with refractory non-small-cell Lung cancer. Nat Med.

[101] Stadtmauer EA, Fraietta JA, Davis MM, Cohen AD, Weber KL, Lancaster E et al. CRISPR-engineered T cells in patients with refractory cancer. Sci (New York NY). 2020;367(6481). 10.1126/science.aba7365 PMC11249135 32029687

[102] Liu X, Zhao Y (2018). CRISPR/Cas9 genome editing: fueling the revolution in cancer immunotherapy. Curr Res Translational Med.

[103] Triebel F, Jitsukawa S, Baixeras E, Roman-Roman S, Genevee C, Viegas-Pequignot E (1990). LAG-3, a novel lymphocyte activation gene closely related to CD4. J Exp Med.

[104] Sierro S, Romero P, Speiser DE (2011). The CD4-like molecule LAG-3, biology and therapeutic applications. Expert Opin Ther Targets.

[105] Jung IY, Kim YY, Yu HS, Lee M, Kim S, Lee J (2018). CRISPR/Cas9-Mediated knockout of DGK improves Antitumor activities of Human T cells. Cancer Res.

[106] Upadhyay R, Boiarsky JA, Pantsulaia G, Svensson-Arvelund J, Lin MJ, Wroblewska A (2021). A critical role for Fas-mediated off-target Tumor killing in T-cell immunotherapy. Cancer Discov.

[107] Tang N, Cheng C, Zhang X, Qiao M, Li N, Mu W et al. TGF-β inhibition via CRISPR promotes the long-term efficacy of CAR T cells against solid tumors. JCI Insight. 2020;5(4). 10.1172/jci.insight.133977 PMC7101140 31999649

[108] Chen J, López-Moyado IF, Seo H, Lio CJ, Hempleman LJ, Sekiya T (2019). NR4A transcription factors limit CAR T cell function in solid tumours. Nature.

[109] Eyquem J, Mansilla-Soto J, Giavridis T, van der Stegen SJ, Hamieh M, Cunanan KM (2017). Targeting a CAR to the TRAC locus with CRISPR/Cas9 enhances tumour rejection. Nature.

[110] MacLeod DT, Antony J, Martin AJ, Moser RJ, Hekele A, Wetzel KJ (2017). Integration of a CD19 CAR into the TCR Alpha Chain Locus streamlines production of allogeneic gene-edited CAR T cells. Mol Therapy: J Am Soc Gene Therapy.

[111] Odé Z, Condori J, Peterson N, Zhou S, Krenciute G. CRISPR-Mediated non-viral site-specific gene integration and expression in T cells: protocol and application for T-Cell therapy. Cancers. 2020;12(6). 10.3390/cancers12061704 PMC7352666 32604839

[112] Sterner RM, Sakemura R, Cox MJ, Yang N, Khadka RH, Forsman CL (2019). GM-CSF inhibition reduces cytokine release syndrome and neuroinflammation but enhances CAR-T cell function in xenografts. Blood.

[113] Kang L, Tang X, Zhang J, Li M, Xu N, Qi W (2020). Interleukin-6-knockdown of chimeric antigen receptor-modified T cells significantly reduces IL-6 release from monocytes. Experimental Hematol Oncol.

[114] Kim MY, Yu KR, Kenderian SS, Ruella M, Chen S, Shin TH (2018). Genetic inactivation of CD33 in hematopoietic stem cells to enable CAR T cell immunotherapy for Acute Myeloid Leukemia. Cell.

[115] Borot F, Wang H, Ma Y, Jafarov T, Raza A, Ali AM (2019). Gene-edited stem cells enable CD33-directed immune therapy for myeloid malignancies. Proc Natl Acad Sci USA.

[116] Humbert O, Laszlo GS, Sichel S, Ironside C, Haworth KG, Bates OM (2019). Engineering resistance to CD33-targeted immunotherapy in normal hematopoiesis by CRISPR/Cas9-deletion of CD33 exon 2. Leukemia.

[117] Gomes-Silva D, Srinivasan M, Sharma S, Lee CM, Wagner DL, Davis TH (2017). CD7-edited T cells expressing a CD7-specific CAR for the therapy of T-cell malignancies. Blood.

[118] Cooper ML, Choi J, Staser K, Ritchey JK, Devenport JM, Eckardt K (2018). An off-the-shelf fratricide-resistant CAR-T for the treatment of T cell hematologic malignancies. Leukemia.

[119] Raikar SS, Fleischer LC, Moot R, Fedanov A, Paik NY, Knight KA (2018). Development of chimeric antigen receptors targeting T-cell malignancies using two structurally different anti-CD5 antigen binding domains in NK and CRISPR-edited T cell lines. Oncoimmunology.

[120] Santomasso B, Bachier C, Westin J, Rezvani K, Shpall EJ. The Other Side of CAR T-Cell Therapy: Cytokine Release Syndrome, Neurologic Toxicity, and Financial Burden. American Society of Clinical Oncology educational book American Society of Clinical Oncology Annual Meeting. 2019;39:433 – 44. 10.1200/EDBK_238691 31099694

[121] Torikai H, Cooper LJ (2016). Translational implications for off-the-shelf Immune cells expressing chimeric Antigen receptors. Mol Therapy: J Am Soc Gene Therapy.

[122] Zhou X (2020). Empowering chimeric antigen receptor T-cell therapy with CRISPR. Biotechniques.

[123] Riolobos L, Hirata RK, Turtle CJ, Wang PR, Gornalusse GG, Zavajlevski M (2013). HLA engineering of human pluripotent stem cells. Mol Therapy: J Am Soc Gene Therapy.

[124] Ren J, Zhang X, Liu X, Fang C, Jiang S, June CH (2017). A versatile system for rapid multiplex genome-edited CAR T cell generation. Oncotarget.

[125] Schlimgen R, Howard J, Wooley D, Thompson M, Baden LR, Yang OO (2016). Risks Associated with Lentiviral Vector exposures and Prevention Strategies. J Occup Environ Med.

[126] Schumann K, Lin S, Boyer E, Simeonov DR, Subramaniam M, Gate RE (2015). Generation of knock-in primary human T cells using Cas9 ribonucleoproteins. Proc Natl Acad Sci USA.

[127] Roth TL, Puig-Saus C, Yu R, Shifrut E, Carnevale J, Li PJ (2018). Reprogramming human T cell function and specificity with non-viral genome targeting. Nature.

[128] Ahmadi M, King JW, Xue SA, Voisine C, Holler A, Wright GP (2011). CD3 limits the efficacy of TCR gene therapy in vivo. Blood.

[129] Legut M, Dolton G, Mian AA, Ottmann OG, Sewell AK (2018). CRISPR-mediated TCR replacement generates superior anticancer transgenic T cells. Blood.

[130] Morton LT, Reijmers RM, Wouters AK, Kweekel C, Remst DFG, Pothast CR (2020). Simultaneous deletion of endogenous TCRαβ for TCR Gene Therapy creates an Improved and Safe Cellular Therapeutic. Mol Therapy: J Am Soc Gene Therapy.

[131] van Hees M, Slott S, Hansen AH, Kim HS, Ji HP, Astakhova K (2022). New approaches to moderate CRISPR-Cas9 activity: addressing issues of cellular uptake and endosomal Escape. Mol Therapy: J Am Soc Gene Therapy.

[132] Gaudelli NM, Komor AC, Rees HA, Packer MS, Badran AH, Bryson DI (2017). Programmable base editing of A•T to G•C in genomic DNA without DNA cleavage. Nature.

[133] Komor AC, Badran AH, Liu DR (2017). CRISPR-Based technologies for the manipulation of eukaryotic genomes. Cell.

[134] Rees HA, Liu DR (2018). Publisher correction: base editing: precision chemistry on the genome and transcriptome of living cells. Nat Rev Genet.

[135] Komor AC, Kim YB, Packer MS, Zuris JA, Liu DR (2016). Programmable editing of a target base in genomic DNA without double-stranded DNA cleavage. Nature.

[136] Nishida K, Arazoe T, Yachie N, Banno S, Kakimoto M, Tabata M (2016). Targeted nucleotide editing using hybrid prokaryotic and vertebrate adaptive immune systems.

[137] Webber BR, Lonetree CL, Kluesner MG, Johnson MJ, Pomeroy EJ, Diers MD (2019). Highly efficient multiplex human T cell engineering without double-strand breaks using Cas9 base editors. Nat Commun.

[138] Broeders M, Herrero-Hernandez P, Ernst MPT, van der Ploeg AT, Pijnappel W (2020). Sharpening the Molecular scissors: advances in gene-editing technology. iScience.

[139] Anzalone AV, Randolph PB, Davis JR, Sousa AA, Koblan LW, Levy JM (2019). Search-and-replace genome editing without double-strand breaks or donor DNA. Nature.

[140] Kim DY, Moon SB, Ko JH, Kim YS, Kim D (2020). Unbiased investigation of specificities of prime editing systems in human cells. Nucleic Acids Res.

[141] Jin S, Lin Q, Luo Y, Zhu Z, Liu G, Li Y (2021). Genome-wide specificity of prime editors in plants. Nat Biotechnol.

[142] Kang SH, Lee WJ, An JH, Lee JH, Kim YH, Kim H (2020). Prediction-based highly sensitive CRISPR off-target validation using target-specific DNA enrichment. Nat Commun.

[143] Ran FA, Hsu PD, Lin CY, Gootenberg JS, Konermann S, Trevino AE (2013). Double nicking by RNA-guided CRISPR Cas9 for enhanced genome editing specificity. Cell.

[144] Trevino AE, Zhang F (2014). Genome editing using Cas9 nickases. Methods Enzymol.

[145] Fu Y, Sander JD, Reyon D, Cascio VM, Joung JK (2014). Improving CRISPR-Cas nuclease specificity using truncated guide RNAs. Nat Biotechnol.

[146] Chew WL. Immunity to CRISPR Cas9 and Cas12a therapeutics. Wiley Interdisciplinary Reviews Systems Biology and Medicine. 2018;10(1). 10.1002/wsbm.1408 29083112

[147] Nowak CM, Lawson S, Zerez M, Bleris L (2016). Guide RNA engineering for versatile Cas9 functionality. Nucleic Acids Res.

[148] Rasul MF, Hussen BM, Salihi A, Ismael BS, Jalal PJ, Zanichelli A (2022). Strategies to overcome the main challenges of the use of CRISPR/Cas9 as a replacement for cancer therapy. Mol Cancer.

[149] Zhao B, Rothenberg E, Ramsden DA, Lieber MR (2020). The molecular basis and Disease relevance of non-homologous DNA end joining. Nat Rev Mol Cell Biol.

[150] Wu J, Zou Z, Liu Y, Liu X, Zhangding Z, Xu M (2022). CRISPR/Cas9-induced structural variations expand in T lymphocytes in vivo. Nucleic Acids Res.

[151] Yin J, Liu M, Liu Y, Hu J (2019). Improved HTGTS for CRISPR/Cas9 off-target detection. Bio-protocol.

[152] Alton E, Armstrong DK, Ashby D, Bayfield KJ, Bilton D, Bloomfield EV (2015). Repeated nebulisation of non-viral CFTR gene therapy in patients with cystic fibrosis: a randomised, double-blind, placebo-controlled, phase 2b trial. The Lancet Respiratory Medicine.

[153] Chen F, Alphonse M, Liu Q (2020). Strategies for nonviral nanoparticle-based delivery of CRISPR/Cas9 therapeutics. Wiley Interdisciplinary Reviews Nanomedicine and Nanobiotechnology.

[154] Xu X, Liu C, Wang Y, Koivisto O, Zhou J, Shu Y (2021). Nanotechnology-based delivery of CRISPR/Cas9 for cancer treatment. Adv Drug Deliv Rev.

[155] Cox DB, Platt RJ, Zhang F (2015). Therapeutic genome editing: prospects and challenges. Nat Med.

[156] Zhang Y, Heidrich N, Ampattu BJ, Gunderson CW, Seifert HS, Schoen C (2013). Processing-independent CRISPR RNAs limit natural transformation in Neisseria meningitidis. Mol Cell.

[157] Ran FA, Cong L, Yan WX, Scott DA, Gootenberg JS, Kriz AJ (2015). In vivo genome editing using Staphylococcus aureus Cas9. Nature.

[158] Kim E, Koo T, Park SW, Kim D, Kim K, Cho HY (2017). In vivo genome editing with a small Cas9 orthologue derived from Campylobacter jejuni. Nat Commun.

[159] Brocken DJW, Tark-Dame M, Dame RT (2018). dCas9: a Versatile Tool for Epigenome Editing. Curr Issues Mol Biol.

[160] Zetsche B, Gootenberg JS, Abudayyeh OO, Slaymaker IM, Makarova KS, Essletzbichler P (2015). Cpf1 is a single RNA-guided endonuclease of a class 2 CRISPR-Cas system. Cell.

[161] Abudayyeh OO, Gootenberg JS, Konermann S, Joung J, Slaymaker IM, Cox DB (2016). C2c2 is a single-component programmable RNA-guided RNA-targeting CRISPR effector. Sci (New York NY).

[162] Meldrum C, Doyle MA, Tothill RW (2011). Next-generation sequencing for cancer diagnostics: a practical perspective. Clin Biochemist Reviews.

[163] Yan W, Herman JG, Guo M (2016). Epigenome-based personalized medicine in human cancer. Epigenomics.

